# Laboratory Findings and Biomarkers in Long COVID: What Do We Know So Far? Insights into Epidemiology, Pathogenesis, Therapeutic Perspectives and Challenges

**DOI:** 10.3390/ijms241310458

**Published:** 2023-06-21

**Authors:** Dimitrios Tsilingiris, Natalia G. Vallianou, Irene Karampela, Gerasimos Socrates Christodoulatos, Georgios Papavasileiou, Dimitra Petropoulou, Faidon Magkos, Maria Dalamaga

**Affiliations:** 1 First Department of Internal Medicine, University Hospital of Alexandroupolis, Democritus University of Thrace, Dragana, 68100 Alexandroupolis, Greece; tsilingirisd@gmail.com; 2 Department of Internal Medicine, Evangelismos General Hospital, 45-47 Ipsilantou Street, 10676 Athens, Greece; natalia.vallianou@hotmail.com; 3 2nd Department of Critical Care, Medical School, University of Athens, Attikon General University Hospital, 1 Rimini Street, 12462 Athens, Greece; eikaras1@gmail.com; 4 Department of Microbiology, Sismanogleio General Hospital, 1 Sismanogleiou Street, 15126 Athens, Greece; gerchristod82@hotmail.com; 5 Department of Biological Chemistry, Medical School, National and Kapodistrian University of Athens, 75 Mikras Asias Street, 11527 Athens, Greece; gpapavasileiou95@gmail.com (G.P.); dimitra.petropoulou@gmail.com (D.P.); 6 Department of Nutrition, Exercise, and Sports, University of Copenhagen, DK-2200 Frederiksberg, Denmark; fma@nexs.ku.dk

**Keywords:** biomarkers, COVID-19, epidemiology, laboratory, long COVID, pathogenesis, post-acute sequelae of SARS-CoV infection (PASC), post-COVID, post-COVID syndrome (PCS)

## Abstract

Long COVID (LC) encompasses a constellation of long-term symptoms experienced by at least 10% of people after the initial SARS-CoV-2 infection, and so far it has affected about 65 million people. The etiology of LC remains unclear; however, many pathophysiological pathways may be involved, including viral persistence; a chronic, low-grade inflammatory response; immune dysregulation and a defective immune response; the reactivation of latent viruses; autoimmunity; persistent endothelial dysfunction and coagulopathy; gut dysbiosis; hormonal and metabolic dysregulation; mitochondrial dysfunction; and autonomic nervous system dysfunction. There are no specific tests for the diagnosis of LC, and clinical features including laboratory findings and biomarkers may not specifically relate to LC. Therefore, it is of paramount importance to develop and validate biomarkers that can be employed for the prediction, diagnosis and prognosis of LC and its therapeutic response, although this effort may be hampered by challenges pertaining to the non-specific nature of the majority of clinical manifestations in the LC spectrum, small sample sizes of relevant studies and other methodological issues. Promising candidate biomarkers that are found in some patients are markers of systemic inflammation, including acute phase proteins, cytokines and chemokines; biomarkers reflecting SARS-CoV-2 persistence, the reactivation of herpesviruses and immune dysregulation; biomarkers of endotheliopathy, coagulation and fibrinolysis; microbiota alterations; diverse proteins and metabolites; hormonal and metabolic biomarkers; and cerebrospinal fluid biomarkers. At present, there are only two reviews summarizing relevant biomarkers; however, they do not cover the entire umbrella of current biomarkers, their link to etiopathogenetic mechanisms or the diagnostic work-up in a comprehensive manner. Herein, we aim to appraise and synopsize the available evidence on the typical laboratory manifestations and candidate biomarkers of LC, their classification based on pathogenetic mechanisms and the main LC symptomatology in the frame of the epidemiological and clinical aspects of the syndrome and furthermore assess limitations and challenges as well as potential implications in candidate therapeutic interventions.

## 1. Introduction

The World Health Organization (WHO) declared COVID-19 a pandemic about 3 years ago, on 11 March 2020. As of 7 June 2023, an estimated 767,750,853 confirmed cases of COVID-19 have occurred, which have resulted in 6,941,095 deaths [1]. Despite the great direct and indirect loss of life [2], accompanied by the considerable societal and financial detrimental consequences of COVID-19 [3], cases of SARS-CoV-2 infection appear to be gradually declining in severity at an individual patient level. Accumulated knowledge regarding COVID-19 pathogenesis and clinical experience that has resulted in improved patient care, increasing levels of collective immunity due to convalescence from natural infection or vaccine coverage and evolving SARS-CoV-2 variant characteristics may have contributed to this phenomenon [4,5]. The predominance of the Omicron variant may be considered a milestone in the course of the pandemic, due to the generally milder features of associated infection in comparison to earlier strains [6]. The currently dominant Omicron XBB.1.5 subvariant also appears to follow this trend [7,8].

With a steadily low mortality rate of SARS-CoV-2 infection, attention is shifted towards survivors who exhibit durable symptoms following apparent clinical recovery [9]. The persistence of clinical manifestations well after successful infection clearance has been described under the umbrella term “long-COVID (LC)”, which covers a wide range of very heterogenous morbid conditions following documented SARS-CoV-2 infection. Even though these disorders are rarely life threatening, they are associated with a considerable detrimental impact on a patient’s quality of life [10], which, combined with the high prevalence of LC, accounts for a large percentage of the overall health care and societal impact associated with the pandemic. According to modest estimates, LC affects at least 10% of infected individuals, which currently translates into roughly 76 million cases globally [11].

The term “long COVID” is used to describe the presence of clinical manifestations and/or complications persisting or manifesting after initial SARS-CoV-2 infection. An overview of the different definitions issued by various sources is presented in Table 1.

Despite the diverse terms and definitions used, there exists a considerable overlap among the different characterizations of LC, particularly in the time frame exceeding beyond 12 weeks after the initial infection. According to the definition of the National Institute for Clinical Excellence (NICE), two fairly distinct conditions may be distinguished: “ongoing symptomatic COVID-19” denotes the presence of symptoms and/or signs persisting over 4 to 12 weeks, while the “post-COVID condition” refers to manifestations appearing during or after SARS-CoV-2 infection persisting for over 12 weeks and which cannot be attributed to a plausible alternative diagnosis [17]. Furthermore, a subset of cases that falls into the definition includes patients suffering prolonged sequelae due to the severity of SARS-CoV-2 infection itself, relating to underlying organ injury during the course of the infection or to hospitalization in an intensive care unit (ICU) setting and associated interventions. All these should be distinguished from the vast majority of cases of LC, in which the duration or intensity of ongoing symptoms is disproportionate to the severity of the initial infection [18].

Not surprisingly, this broad definition includes conditions with a great variety of clinical manifestations ranging from constitutional, non-specific physical symptoms to signs of isolated organ dysfunction, including neuropsychiatric disorders [11,19,20]. Likewise, many putative mechanisms have been implicated in the pathogenesis of LC, including viral persistence, endothelial dysfunction, coagulation disorders and microthrombotic manifestations, the disruption of gut microbiota, neuroinflammation, autoimmunity and mast cell activation, the reactivation of dormant viral pathogens and perturbations of the brainstem and autonomic nervous signaling, among others [11,21,22,23]. Accordingly, a wide range of non-specific laboratory findings have also been associated with LC, albeit with few indexes showing promise as candidate biomarkers for the identification of possible cases [11,24].

There is an urgent need to pinpoint patient-level risk factors and characteristics in order to successfully identify patients at a high risk of developing LC and to properly diagnose this syndrome. Unfortunately, efforts towards that end are hampered by the diversity of presumed organ involvement and related clinical manifestations, limited sample sizes of studies from which the corresponding primary data have been derived and small effect sizes of ascertained associations. At present, there are only two reviews (one scoping and one systematic) summarizing relevant biomarkers. However, these reviews do not cover the entire current umbrella of biomarkers till May 2023, detailed discussions of their link to etiopathogenetic mechanisms, the diagnostic work-up in a comprehensive manner, comparisons with acute severe COVID-19, the provision of extensive data on epidemiologic data and risk factors, clinical manifestations, discussions on the resemblance with post-viral syndromes or therapeutic perspectives [25,26]. Herein, we aim to review the available evidence on the typical laboratory manifestations and candidate biomarkers of LC, their classification based on etiopathogenetic mechanisms and the main LC symptomatology in the frame of the epidemiological and pathogenetic aspects of the syndrome and furthermore assess potential implications in candidate therapeutic interventions.

## 2. Epidemiology of Long COVID and Risk Factors

The exact frequency of LC is challenging to determine, due to the non-specific nature of its clinical manifestations, the absence of consequent screening and the lack of diagnostic markers, which likely result in under-reporting. Furthermore, several reports are confounded by the absence of a comparator control group free of recent SARS-CoV-2 infection, which makes a precise estimate of COVID-attributable symptoms unfeasible. According to modest estimates, around 10% of non-hospitalized survivors experience persistent symptoms [11], while percentages are considerably higher among those hospitalized, with more than 70% reporting at least one compatible clinical manifestation [27]. These numbers may not reflect the true healthcare and societal impact of LC by themselves, if the high incidence of SARS-CoV-2 infection on one hand and the relative long duration (often, several months) of post-infectious manifestations on the other [28] are not taken into account. This likely results in a considerably higher overall true prevalence of LC-associated debilitating symptoms in the general population [29]. Furthermore, although LC is considered a spectrum of chiefly benign disorders with a principal impact on quality of life, rather than loss of life, it cannot be definitely excluded that the conditions falling into its spectrum may contribute to a fraction of excess COVID-19-attributable death. Between 1 January 2020 and 30 June 2022, a total of 3544 of death certificates in the United States listed LC as an underlying or contributing cause of death [30]. Although this number represented only 0.3% of COVID-related deaths, it likely represents an underestimation of actual figures due to inadequate reporting, especially within specific ethnic subgroups. Nevertheless, based on current evidence, there is no definite proof that LC directly leads to death, and its implication in excess COVID-related deaths remains hypothetical. Even in the aforementioned cases, LC may have existed as one of many ongoing comorbidities.

It is noteworthy that the emergence of new variants and the subsequent evolution of the features of individual infections as well as the pandemic itself as a whole are accompanied by a shift in the epidemiological features of LC [31]. With respect to the Omicron variant, the subvariants of which are currently dominant on a worldwide scale, persistent symptoms following infection are likely of a lower severity and shorter duration compared with the earlier wild-type, Alpha, Beta and Delta variants [29,32], being also of lower overall relative prevalence compared with non-infected controls [33]. A recent pooled analysis of two population-based cohorts has shown that the risk of LC is lower after infection with the Omicron variant and following vaccination [34]. Interestingly, the incidence and severity of the Multisystem Inflammatory Syndrome in Children (MIS-C) decreased during the Omicron wave of the pandemic in comparison to earlier waves [35]. It is currently unclear if this observation is primarily a consequence of the altered natural course of infection caused by the Omicron variant or simply confounded by the effects of increasing vaccine coverage (see below) or the increasing prevalence of reinfections and breakthrough infections in the Omicron era [36,37,38,39].

Although most epidemiological data have been collected from adult populations, children of all ages and adolescents are considerably more likely to report chronic symptoms in the spectrum of LC after documented SARS-CoV-2 infection compared with non-infected controls of similar ages [40,41]. Uninfected control pediatric populations report several LC-compatible symptoms with a considerably higher frequency than adult controls [42], which renders a direct comparison regarding LC prevalence between children/adolescents and adults challenging. Of note, post-COVID neurodevelopmental sequelae may also be encountered in infants of mothers infected during pregnancy in the first year of life [43]. Furthermore, certain pathogenetic features (e.g., inflammatory, immunological, pertinent to endothelial dysfunction), as well as diverse clinical manifestations of LC, may overlap with those of the Multisystem Inflammatory Syndrome in Children (MIS-C), which was originally described in pediatric populations in the early post-infection period [44]. According to the case definition of MIS-C [45], it is certain that a proportion of affected patients also fall into the category of LC, at least in the “ongoing symptomatic COVID-19” category. Semantics aside, this could harbor implications for the better understanding not only of LC but also of the pathophysiology of MIS-C, which remains largely elusive, while also guiding potential therapeutic interventions for both conditions. Nevertheless, a presumed pathogenetic connection between the two conditions cannot be substantiated based on available data and remains speculative.

Apart from the aforementioned impact of different viral variants, there are a number of patient-level risk factors, which have been demonstrated to confer a higher risk for LC. According to a recent meta-analysis of 41 studies, among the population of COVID-19 survivors, an older age (Odds Ratio (OR): 1.21), female sex (OR: 1.56), higher Body Mass Index (BMI) (OR: 1.15), smoking (OR: 1.10), the presence of physical and mental comorbidities (OR: 2.48), and hospitalization or admission in the ICU (OR: 2.38) are associated with a higher risk of LC [46]. A longer hospital stay [47], a Black or Hispanic ethnic background and a low socioeconomic status have also been associated with an increased risk [11,48]. In pediatric and young adult populations, an age of less than 5 years, an age greater than 10 years, female sex, Black or Hispanic race, the presence of chronic comorbidities, acute-phase hospitalization, infection with a pre-Omicron variant and ambient long-term air pollution (e.g., exposure to particulate matter ≤ 2.5 μm) have been implicated as risk factors [49,50,51]. Particularly, children with LC were more likely to have suffered from attention deficit hyperactivity disorder, chronic urticaria or allergic rhinitis before SARS-CoV-2 infection [52].

Regarding the impact of prior immunization, and although relevant data from available studies are not unequivocal, the risk for LC after infection following vaccination is likely lower than that of unvaccinated individuals [36]. Analysis of a random sample of adults in the United Kingdom revealed a 41% lower risk of LC following infection among those that had received two vaccine doses (either mRNA-based, adenovirus vector or combined), compared with those who received no vaccination [53]. Data from the US Department of Veterans Affairs national healthcare database demonstrated a more modest risk reduction of 15% [54]. When focusing on the prevention of specific symptoms, such as brain fog or muscle pain, the observed effects may be of a greater magnitude [55]. Interestingly, the effect of vaccination in individuals already diagnosed with LC is variable, with 61.9% reporting no change, 16.7% amelioration and 21.4% a worsening in symptoms, suggesting that active immunization following native infection may not be generalizable as a secondary prevention strategy among affected individuals [56]. Regarding reinfections and the severity of COVID-19, a recent meta-analysis has found no significant difference in the clinical pattern and severity of infection between primary infection and reinfection [57]. However, available data with respect to LC risk following reinfection in both vaccinated and unvaccinated individuals are scarce, although existing evidence points towards an increased risk for LC after repeated infection [58].

## 3. Clinical Manifestations of Long COVID

A wide range of non-specific clinical symptoms and manifestations from virtually every organ and system have been reported as long-term sequelae of SARS-CoV-2 infection. General systemic symptoms (chronic fatigue, arthralgia/myalgia, sleep disorders) are frequently reported. In a meta-analysis, fatigue is the most common symptom experienced [59]. The most frequent post-COVID symptoms in children and adolescents are fatigue, lack of concentration and myalgias [60,61].

As COVID-19 is primarily a respiratory infection, symptoms related to the respiratory system are common, such as chronic cough, breathlessness, exertional dyspnea and chest tightness, which occasionally—but not always—reflect a chronic post-infectious respiratory pathology, with abnormal lung function tests and/or radiological changes [62,63,64]. Upper-airway-related symptoms (voice changes, hoarseness, rhinorrhea, impaired hearing, loss of smell/taste) are also reported. Disorders related to the cardiovascular system (exercise intolerance, chest pain, palpitations, thrombotic manifestations) are also frequent and potentially pathogenetically related to the dysfunction of the cardiovascular autonomic system. Gastrointestinal manifestations (appetite loss, constipation, nausea, abdominal pain, acid reflux) may parallel perturbations of the gut flora or be related to prolonged intestinal viral shedding. Most prominently, neuropsychiatric disorders are considered typical features of the LC spectrum, ranging from mental fatigue, inability to concentrate and poor memory (collectively referred to as “brain fog”) to major depression, anxiety and post-traumatic stress disorder. Skin rashes and hair loss are frequently reported [11,65].

The variety and heterogeneity of LC clinical manifestations, as well as their non-specific nature, contribute to the challenge of the systematic study of LC as a uniform syndrome. Nonetheless, it is likely that groups or clusters of symptoms exist (namely, central neurological, cardiorespiratory, systemic/inflammatory and abdominal clusters), which are more likely to affect individuals as a group rather than as a constellation of chaotic manifestations independently of each other [66]. Interestingly, specific symptom clusters appear to be associated with LC following infection by different SARS-CoV-2 variants [66]. This observation may have important implications not only for the identification of patient groups likely to benefit from specific therapeutic interventions but also for deciphering the underlying pathogenetic mechanisms that drive LC development.

The range of subjective symptoms reported in conjunction with LC may or may not be associated with objectively proven underlying organ damage. This is an important consideration, since the latter may constitute a significantly more objective prognosis-determining factor than the self-report of the clinical symptom. Objective and lasting organ involvement is not uncommon in the context of LC, with 69% and 59% of affected patients exhibiting the dysfunction of at least one organ 6 and 12 months following SARS-CoV-2 infection, respectively, while the corresponding frequencies for multiorgan involvement are 23% and 27%, respectively [67].

One of the earliest recognized features of LC is the dysfunction of the autonomic nervous system, a condition with unknown pathogenesis, duration and prognosis, manifesting itself with signs of dysregulated system functions, primarily of the cardiovascular autonomic nervous system, most prominently postural orthostatic tachycardia, inappropriate resting sinus tachycardia and exercise intolerance [68,69]. Before the COVID-19 era, the bulk of evidence linking cardiac autonomic neuropathy with a worse cardiovascular prognosis and propensity towards life-threatening cardiac arrhythmias was derived from observations in patients with diabetes mellitus (DM) [70]. Likewise, it would be plausible that autonomic dysfunction is a major driver of ongoing cardiovascular symptoms in LC [71], contributing to the increased cardiovascular risk and worse prognosis of affected patients [72].

COVID-19 shares a bidirectional relationship with type 1 and type 2 DM [73]: On the one hand, pre-existing DM predisposes patients to a more severe and complicated infection course [39,74,75,76,77]. On the other hand, a higher risk of newly diagnosed type 1 or type 2 DM has been demonstrated among survivors of SARS-CoV-2 infection [73,78]. It can be assumed that, at least in the case of type 2 DM, a proportion of new diagnoses may represent incidental discoveries of undiagnosed pre-existing DM cases emerging through the contact of infected patients with healthcare services. However, the underlying mechanisms driving the association between SARS-CoV-2 infection and the emergence of DM are unclear. In the case of type 1 DM, the triggering of an autoimmune process directed against pancreatic beta cells may be assumed [79]; otherwise, there is evidence of a direct adipocyte viral infection and subsequent adipose tissue dysfunction, resulting in insulin resistance (IR) and the promotion of overt hyperglycemia and type 2 DM [80].

## 4. Are Clinical Manifestations and Pathogenesis Unique to SARS-CoV-2 Infection?

As the spectrum of LC manifestations is becoming increasingly understood among clinicians and researchers, the question arises whether this is a unique feature of SARS-CoV-2 infection itself or whether it shares resemblance with similar syndromes observed following respiratory—or other—viral illnesses. Undoubtedly, the magnitude and sheer number of cases during the last three years allowed for the better study and characterization of this syndrome in conjunction with COVID-19. Nevertheless, chronic post-viral syndromes have been described in conjunction with earlier coronavirus outbreaks. Chronic fatigue was reported by 60% of SARS survivors 12 months post-recovery [81] and as many as 40% of survivors ~41 months following the acute infection [82]. Similar symptoms were reported among 48% and 33% of Middle East Respiratory Syndrome (MERS) survivors 12 and 18 months after the initial illness, respectively, in conjunction with depressive and post-traumatic stress disorder features [83].

Many of those with post-SARS syndrome fulfilled the definition of the International Chronic Fatigue Syndrome Study Group for Myalgic Encephalomyelitis/Chronic Fatigue Syndrome (ME/CFS) [82,84,85]. Apart from chronic fatigue, poor cognitive function, a shortness of breath, functional gastrointestinal disorders and palpitations are other typical features [86]. Furthermore, features of ME/CFS have been described in conjunction with other epidemic/pandemic (H1N1 influenza virus [87], Ebola virus [88], West-Nile virus [89]) and non-epidemic (most commonly, by *Herpesviridae*: EBV, CMV, VZV, HHV-6, HHV-7) [90] viral illnesses. Interestingly, the prevalence of ME/CSF globally is approximately 1% (17 to 24 million affected individuals) [91]. It seems plausible that a considerable proportion of individuals affected by LC would fulfill formal criteria for ME/CFS and furthermore that a certain fraction of LC cases corresponds to a post-viral syndrome not specific to SARS-CoV-2 infection, although further research into the underlying mechanisms of this condition is needed to support this hypothesis.

## 5. Pathogenesis of Long COVID

**Figure 1:**
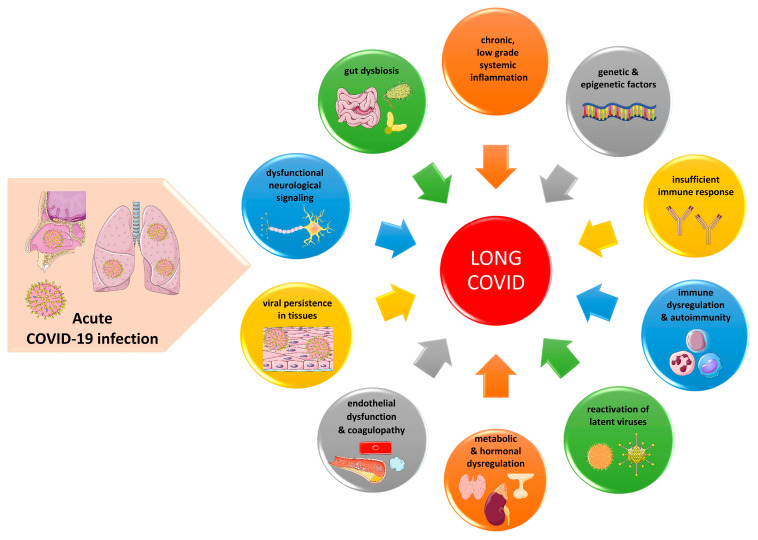
Potential pathophysiological mechanisms involved in the development of long COVID following acute SARS-CoV-2 infection. (All images were constructed with the free medical site http://smart.servier.com/ (accessed on 15 May 2023) by Servier licensed under a Creative Commons Attribution 3.0 Unported License.)

The underlying pathogenetic mechanisms in LC are not fully elucidated; however, there are several interacting pathways that may contribute to the symptomatology of LC, as summarized in Figure 1. Overall, the following mechanisms are implicated in the pathobiology of LC:

(1) A chronic, low-grade inflammatory response is associated in several cases with the severity of ongoing symptoms particularly in patients who were hospitalized [92]. This hyperinflammatory response may be facilitated partly by mast cell activation, attributed to the loss of genetic regulation of mast cells by SARS-CoV-2 [93,94].

(2) Immune dysregulation is linked to decreased B cell and T cell numbers, increased innate immunity and persistent SARS-CoV-2 shedding, which further contribute to the chronic inflammatory response and immune activation in LC [95,96].

(3) SARS-CoV-2 (spike protein and viral mRNA) may persist in certain patients with LC in cryptic tissue reservoirs following acute COVID-19, triggering repeated and sustained immune responses leading to chronic symptoms [97,98]. The spike protein, which represents one of the main virulence factors of SARS-CoV-2, is a significant viral protein for the attachment of the virus to target cells bearing the angiotensin-converting 2 enzyme (ACE-2) surface receptors. Moreover, the spike protein may be released by the host cell via extracellular vesicles (EVs) and reach distant tissues and organs through the circulation [99].

Viral persistence is observed in certain viruses, such as herpesviruses (Herpes Simplex Virus (HSV), Varicella-Zoster Virus (VZV), Epstein–Barr Virus (EBV), Cytomegalovirus (CMV)), Human Immunodeficiency Virus (HIV), hepatitis viruses (HBV and HCV) and human papillomavirus (HPV), which may not be cleared from the host after the initial phase remaining in a chronically lytic and/or latent conformation in the host [100].

(4) Insufficient immune responses and antibody production against SARS-CoV-2 in the acute phase of COVID-19 infection may be associated with viral persistence [101]. Interestingly, immunologic imprinting, where pre-existing immune responses against related coronaviruses, such as 229E, HKU1, NL63 and OC43, as well as defective immune responses against SARS-CoV2 in the cerebrospinal fluid (CSF), may lead to incomplete virus clearance from the brain, persistent neuroinflammation and neurological post-acute sequelae of COVID-19 [102].

(5) The reactivation of latent viruses, such as EBV, VZV, HSV and HHV-6, during and after acute COVID-19 infection in various organs may lead to a plethora of chronic symptoms after COVID-19 (e.g., fatigue, myalgia, cognitive disorders) [103]. These viruses have been implicated in several disorders, including rheumatoid arthritis, multiple sclerosis, lymphomas and cancers [104,105,106,107,108,109].

(6) SARS-CoV-2 may trigger autoimmunity via molecular mimicry, epitope spreading and the activation of antigen-presenting cells (APCs), following the example of other viruses and bacteria, such as HCV, enteroviruses (coxsackievirus B), EBV, *Helicobacter pylori*, *Yersinia enterocolitica*, etc. [110,111,112,113,114]. Immunodominant epitopes of SARS-CoV-2 (e.g., nucleocapsid protein, spike protein) may share homology with host proteins providing insight into viral pathogenesis following COVID-19 [115,116].

(7) SARS-CoV-2 infection could cause gut dysbiosis, altering the balance of gut microbiota, also acting as a bacteriophage, causing epithelial permeability and the translocation of pathogens into the circulation [117,118]. Gut dysbiosis could additionally be implicated in viral persistence, chronic inflammation, immune dysregulation and endothelial dysfunction [94,119,120,121,122,123].

(8) LC is characterized by a persistent endothelial dysfunction and a hypercoagulable state [124]. SARS-CoV-2 may also affect the endothelial cells throughout a plethora of tissues, altering cell morphology and leading to the apoptosis of endothelial cells. For example, in the brain, the infection of the microvascular endothelium in the subcortical white matter is documented by the presence of microscopic hemorrhagic and ischemic lesions, while, in the heart, there is edema of endothelial cells in small arterioles, venules and capillaries as well as the necrosis of individual myocytes [125,126]. The attachment of activated neutrophils in capillaries and their subsequent obstruction in several organs including the heart, brain and lungs in conjunction with the presence of fibrin amyloid microclots, which provoke tissue hypoxia and oxidative stress, may be implicated in the symptomatology of LC [94,127]. In turn, oxidative stress stimulates the generation of proinflammatory cytokines, establishing a vicious cycle [128,129,130,131]. The presence of fibrin aggregates or microclots and activated thrombocytes has also been documented in the blood of patients suffering from type 2 DM, acute COVID-19 infection and ME/CFS contributing to the chronic inflammatory and thrombotic phenomena of these entities [132,133,134].

(9) LC is also associated with hormonal and metabolic dysregulation, characterized by a dysfunction of the hypothalamus–pituitary–adrenal axis [135]. This dysregulation may be caused by hypophysitis, hypothalamic damage, neuroinflammation or the molecular mimicry of some viral aminoacids that mimic ACTH residues [135,136]. Moreover, IR in conjunction with activated immune-inflammatory, oxidative and nitrosative stress pathways may be implicated in neurotoxicity and depressive symptoms observed in LC [137]. IR may be associated with lower metabolic activity in the medial prefrontal cortex and hippocampal volume, neurocognitive impairments, defective synaptic plasticity and neurodegeneration [138]. Interestingly, metabolic dysfunction (i.e., obesity, IR, metabolic syndrome and type 2 DM) constitute predisposing risk factors for severe acute COVID-19, while recent data have underscored that the combination of metabolic dysfunction, chronic immune activation and viral persistence in adipose tissue could exacerbate or predispose to LC [139]. Finally, deficiency in melatonin, a sleep hormone with properties that enhance the adaptive immune response, has been associated with a higher risk for COVID-19 [140]. It could also be a predisposing factor in LC as observed in other viral infections and chronic inflammatory disorders [141].

(10) Cognitive impairment and neurological symptoms in LC may be attributed to the delayed clearance of SARS-CoV-2, endothelial dysfunction and microclot formation, neuronal injury, neuroinflammation, blood–brain barrier disruption with infiltrating polyreactive autoantibodies, microglial reactivity or impaired neurogenesis [11,55,142,143,144]. Autonomic nervous system dysfunction and symptoms consistent with dysautonomia, such as orthostatic hypotension, tachycardia, fatigue, temperature dysregulation, etc., could be due to the dysfunctional signaling in the brainstem and/or vagus nerve; the presence of autoantibodies against the G-protein-coupled adrenergic receptor and muscarinic acetylcholine receptor; small fiber neuropathy; neuroinflammation; the failure of peripheral vasoconstriction associated with relative hypovolemia; or cerebral hypoperfusion [94,145,146]. To date, very limited evidence exists on the pathophysiological mechanisms implicated in the neurological symptoms of LC; therefore, all the abovementioned mechanisms are hypothetical. It should be noted that a direct CNS infection by SARS-CoV-2, although possible [98,147], is likely quite uncommon [148] and hence unlikely to account for a significant fraction of neurological manifestations of LC.

(11) Mitochondrial dysfunction may be an attractive explanation for the pathogenesis of LC, particularly for symptoms such as persistent fatigue and exercise intolerance. Current evidence has documented indices of mitochondrial dysfunction in LC, including dysregulated mitochondrial metabolism, a loss of mitochondrial membrane potential, an altered fatty acid metabolism with dysregulated mitochondrial lipid catabolism, and redox imbalance [149,150]. Mitochondrial dysfunction, lactate accumulation during exercise challenges, and defective oxygen extraction and delivery to target organs may also be associated with exercise intolerance in LC [149,150,151,152,153,154]. However, mitochondrial dysfunction could also be a consequence of cellular hypoxia, elevated oxidative stress, immune dysregulation or increased inflammation.

## 6. Laboratory Findings and Biomarkers in Long COVID

The main laboratory findings and biomarkers in acute severe COVID-19 encompass biomarkers of systemic inflammation, including proinflammatory cytokines, chemokines and complement proteins as well as biomarkers of endothelial injury and coagulation cascades, including markers of platelet activation and neutrophil extraction trap formation [124,155]. In particular, the cardinal laboratory features with prognostic potential are as follows: lymphopenia, either isolated or in parallel with an increased absolute neutrophil count; elevated concentrations of C-reactive protein (CRP), interleukin (IL)-6 and IL-2R; and increased levels of lactate dehydrogenase (LDH), D-dimer, ferritin, hepatic function markers and troponins [156,157,158]. Besides the elevation of D-dimer, other biomarkers of coagulation abnormalities, such as the prolongation of PT and aPTT, the presence of severe thrombocytopenia and the elevation of fibrin degradation products, may reflect life-threatening disseminated intravascular coagulation (DIC), which requires increased vigilance and prompt intervention [159,160,161,162]. All these biomarkers are associated with an increased risk of disease worsening, including acute myocardial infarction, venous thromboembolism and acute ischemic stroke [124,155]. On the contrary, venous thromboembolic disorders, deep vein thrombosis and pulmonary embolism are not primary characteristics of LC. Table 2 synopsizes the main differences in risk factors and clinical and laboratory characteristics between acute severe COVID-19 and LC.

Due to the multifactorial nature of LC, it is important to mention that there are no specific tests that can be used for its diagnosis. Likewise, clinical features as well as laboratory findings and biomarkers may not be safely attributed to LC. Therefore, the diagnostic evaluation should be personalized while at the same time reassuring the patient that LC is a clinical entity with a broad spectrum of clinical manifestations. Occasionally, clinicians may a priori tend to attribute manifestations related to POTS or ME/CFS to mental health disorders, such as anxiety or depression disorders [11,163]. It has been recognized that COVID-19 may act as a trigger for anxiety or depressive symptoms or even lead to post-traumatic stress disorder in certain cases [164,165]. Although it cannot be excluded that a fraction of symptoms in the spectrum of LC could represent psychiatric manifestations, vigilance from the clinician’s side is crucial, in order to avoid the misdiagnosis of potentially important and prognosis-defining underlying conditions for mental disorders.

The initial diagnostic work-up should take into account that persisting clinical manifestations belong to the spectrum of LC and not to an underlying disorder. Table 3 presents a general laboratory and imaging diagnostic approach to help clarify symptom etiology, rule out other underlying disorders and evaluate the clinical manifestations and resolutions of some features of LC.

Besides the usual hematologic, biochemical, coagulation and inflammatory markers, including CRP and serum ferritin, the diagnostic approach may comprise cardiac markers, hormonal and vitamin markers, glycemic indices, such as glucose or hemoglobin A1c, and the determination of autoantibodies. These basic tests often return results within the normal reference range in patients with LC; however, some inflammatory biomarkers may persist for longer [11,92]. The imaging diagnostic approach is important for the evaluation of pulmonary fibrosis resolution with a repeated chest CT scan); cardiac function with a cardiac ultrasound; neurologic and cognitive disorders with brain magnetic resonance imaging, etc. [166]. It is important to mention that some diagnostic tools may be used for the evaluation of features of LC, such as the validated “symptom burden questionnaire for LC” (SBQ-LC), tests examining orthostatic intolerance for the postural orthostatic tachycardia syndrome (POTS), cardiac magnetic resonance for cardiovascular abnormalities and residual pathology, an electrocardiogram depicting novel QRS fragmentation due to cardiac injury, pulmonary function tests, etc. [166,167,168,169]. Nevertheless, the majority of diagnostic tools for LC are under development, such as the use of hyperpolarized magnetic resonance to identify abnormalities of pulmonary gas exchange, imaging to detect microclots or small fiber neuropathy [11,170], while some other tests were also used in patients with ME/CFS and dysautonomia, such as the determination of serum or salivary cortisol, antibodies against herpesviruses, total immunoglobulin concentrations (IgG, IgA, IgM, IgG3), the test for natural killer cell function, tests for orthostatic intolerance, etc. [171]. It is of paramount importance to develop and validate biomarkers for the prediction, diagnosis and prognosis of LC and its therapeutic response. In the following subsections and Table 4, we discuss some promising biomarkers for the prediction, diagnosis and prognosis of LC as well as biomarkers associated with clinical manifestations of LC.

### 6.1. Biomarkers of Systemic Inflammation

In response to a viral infection at the acute phase, the immune system activates several cells (T cells, macrophages, etc.), releasing different inflammatory molecules including cytokines and chemokines. SARS-CoV-2 infection may trigger a “cytokine storm” characterized by a moderate proinflammatory cytokine release associated with severe monocyte dysregulation [217]. Acute respiratory distress syndrome (ARDS), one of the leading causes of death in patients with COVID-19, is mainly triggered by the cytokine storm [218]. Interestingly, varied and relapsing symptoms of LC may be due to increased cytokines, released by an abnormal immune response [219].

In the past 2 years, there have been several case-control, prospective and retrospective studies that have investigated inflammatory biomarkers in LC, yielding mixed results. An early meta-analysis of 15 studies on the prevalence of long-term effects in LC, including laboratory abnormalities, reported limited evidence for systemic inflammation after approximately 4 months post-infection [20]. In particular, 8% of patients had elevated CRP and ferritin, while 3% and 4% of patients had increased levels of IL-6 and procalcitonin, respectively [20]. However, this was an early meta-analysis that included a very small number of studies examining laboratory abnormalities with a moderate number of participants. In contrast, a very recent meta-analysis of 23 studies (14 prospective and 9 retrospective case control) with 18 meta-analyzed biomarkers has found that survivors of LC presented higher levels of CRP, D-dimer, lactate dehydrogenase (LDH) and leukocytes than controls without LC; however, the effect sizes were small [172]. After sensitivity analyses, lymphocytes and IL-6 were also significantly elevated in cases with LC. In particular, differences in the levels of D-dimer, LDH and lymphocytes were prominent in the group of patients with organ abnormalities assessed by imaging and functional studies, while differences in levels of IL-6 were observed in the group of patients with a persistence of symptoms [172]. Furthermore, differences in the levels of LDH, leukocytes and N-terminal pro b-type natriuretic peptide (NT-Pro-BNP) were found in the group of patients with symptoms of less than 6 months, while differences in D-dimer levels were found in patients with symptoms of more than 6 months. IL-8, a chemokine activating neutrophils at the inflammation site, was found elevated in LC patients compared to healthy controls based on only two studies [172]. Finally, another meta-analysis of 22 case-control observational studies has found that IL-6 was higher in patients with LC compared with healthy individuals and those without post-acute sequelae of COVID-19 but lower than in patients during the acute phase of COVID-19 [173]. IL-6, which is a practical biomarker of systemic inflammation and adverse outcomes of acute COVID-19, may serve as a useful predictor of LC after a time window of 4-weeks post-infection, informing on the “early stage” of LC [173]. In several studies, higher levels of IL-6 were sustained for up to 7 months in patients with LC [92,178,220,221]. Likewise, in some studies, CRP, the acute phase protein of hepatic origin that is elevated following the release of IL-6 by macrophages and T cells, is persistently increased in LC patients from the early phase [222] and up to 7 months after [25,204].

Additional studies on cytokines and chemokines have shown an augmentation of IL-2 (which stimulates the growth of helper, cytotoxic and regulatory T cells), IL-17 (which is associated with inflammation and autoimmunity), interferon (IFN)-γ (which is critical for innate and adaptive immunity against viral infections), CCL3 and CCL5 in the early phase of LC [25,178,220,223,224]. Patients experiencing LC with cognitive symptoms have shown elevated CCL11, which is associated with the inhibition of neurogenesis, aging and cognitive function, compared to those with LC but without cognitive symptoms [225,226]. Contradictory results were reported on anti-inflammatory cytokines IL-4 and IL-10, which were found to be reduced or elevated in LC patients [178,224]. Compared to recovered patients, IFN-γ remained elevated at 2 months, tumor necrosis factor-α (TNF-α) at 4 months and IFN-β and IFN-λ1 at 11 months in LC patients [96,220]. Interestingly, the genotype AA of the *IFNG* gene was more frequently found in LC patients [227]. Other acute phase proteins that respond to proinflammatory cytokines and rise following inflammation and tissue injury, such as serum amyloid 1 (SAA1) and SAA4, were increased in the microclots of patients with LC at 3 months [204].

Overall, certain phenotypes of LC are associated with increased biomarkers of systemic inflammation; however, data are limited. These biomarkers may present a predictive value to detect patients at risk of LC as well as a diagnostic value for certain LC phenotypes. Further longitudinal studies are required to observe if the pattern of the elevation of certain cytokines follows a similar pattern seen in ME/CSF, where some cytokines decrease after being elevated in the first years of the disease despite the persistence of symptoms [228].

### 6.2. Immune Profiling in Long COVID

Studies investigating immune dysregulation in patients with LC have found a plethora of alterations in immune cells. Increased inflammatory monocytes (CD14^+^, CD16^+^, CCR5^+^) were found in cases before the development of LC and in the convalescent chronic period [178] along with elevated non-classical monocytes, which are also associated with various chronic inflammatory and autoimmune conditions [174]. Studies have also documented increased natural killer (NK) cells expressing markers of memory and activation, higher plasmacytoid dendritic cells exhibiting CD86 and CD38 markers, which play an important role in antiviral immunity and have been implicated in the initiation and development of many autoimmune and inflammatory diseases, and decreases in the numbers of conventional dendritic cells [96,174,176].

Interestingly, individuals with LC present B and T cell abnormalities persisting for at least one year, including decreased naive B and T cells; increased B cells and double negative B cells, which expand in older individuals but also accumulate prematurely in autoimmune and infectious diseases; decreased CD4^+^ and CD8^+^ effector memory cells; increased or decreased SARS-CoV-2 CD8^+^ T cells expressing cytotoxic markers in patients with respiratory symptoms or gastrointestinal symptoms, respectively; reduced exhausted lymphocytes (CD4^+^/CD8^+^ expressing PD1) before clinical manifestations of LC; and increased exhausted lymphocytes in the convalescent period of LC [25,96,174,176,190,229].

No firm conclusions can be drawn about T regulatory (Treg) cells’ alterations in LC due to inconclusive data [175]. After their initial infection with SARS-CoV-2, patients with LC manifest a dysregulation of Treg cell function. These cells play a crucial role in self-tolerance by inhibiting T cell proliferation and cytokine production and preventing autoimmunity. However, there are contradictory findings with higher and lower frequencies of Treg amid CD4^+^ cells in cases with LC compared to recovered subjects [176,177], while a lower Treg proportion was found in 121 patients with LC compared to controls [178].

Overall, although there is no specific immune signature due to the heterogeneity of LC, the abnormalities in the immunophenotype of cases with LC underscore a prolonged antiviral immune response, which is common during chronic exposure to viral antigens and viral persistence.

### 6.3. Biomarkers Reflecting SARS-CoV-2 Persistence

Several studies with a small number of participants as well as case series and reports have found components of viral persistence, which may trigger symptoms of LC, particularly gastrointestinal symptoms, such as viral proteins (spike and nucleocapsid) and/or SARS-CoV-2 RNA in feces, plasma, urine, the brain, muscles, eyes, lymph nodes, cardiovascular organs, the liver and lung tissue [98,179,180,181,183,230]. A histopathological study of infected tissues via performing autopsies on 44 COVID-19 cases has found SARS-CoV-2 RNA broadly distributed in 84 distinct anatomical sites up to 230 days after infection. Interestingly, notwithstanding that viral RNA was indetectable in plasma amid all deceased cases, viral persistence was identified throughout a plethora of tissues using high-sensitivity droplet digital PCR, suggesting the sustained presence of a low viral load in biospecimens of COVID-19 patients [98]. Moreover, intestinal endoscopic studies, particularly in patients with inflammatory bowel disease (IBD), have shown a SARS-CoV-2 presence in the gut epithelium or stools even after 6 months post-infection, suggestive of a potential viral reservoir triggering sustained inflammatory responses in some cases of LC [180,182,231]. Interestingly, detectable SARS-CoV-2 RNA in stool samples and increased circulating spike, S1 and nucleocapsid (N) antigens were found in children with MIS-C compared to children with acute COVID-19 or controls, suggesting that viral persistence may trigger the hyperinflammatory responses defining MIS-C [183]. Nonetheless, Sigal et al. did not show any persistence of the plasma spike antigen in a multicentric cohort study of children with MIS-C employing an ultra-sensitive electro-chemiluminescent immunoassay [232].

Overall, current available evidence has highlighted that the duration of SARS-CoV-2 infection in cases may persist longer than determined by PCR-negative tests on nasopharyngeal swabs or bronchoalveolar lavage fluids. Sustained low-grade multisystem inflammation in both adults and children may be attributed to a lingering SARS-CoV-2 infection or reinfection [233]. The use of antiviral agents against SARS-CoV2, such as nirmatrelvir with ritonavir, could eradicate viral reservoirs and attenuate symptoms of LC.

### 6.4. Humoral and Cellular Response against SARS-CoV-2 in Long COVID

Adaptive immune responses are a key component for the control of viral infection, the severity of the infection and the protection from reinfection. During acute infection, immune responses are crucial for establishing the coordinated immune response required for the development of antibody and memory B cells. Clinical trials have documented that neutralizing antibodies to SARS-CoV-2 may decrease the severity of disease, mortality and length of hospitalization [234].

Conflicting results have been reported regarding anti-SARS-CoV-2 antibody responses and the occurrence of LC with both positive, neutral and negative associations of SARS-CoV-2-specific antibody titers against spike, S, the receptor-binding domain (RBD) domain of the spike protein, or N protein in both hospitalized and non-hospitalized patients [101,174,184,185,186,187].

T cells play a critical role in COVID-19 immunity, severity and mortality, yet relatively little is known about their contribution to LC. A limited set of studies that have investigated SARS-CoV-2-specific T cell responses have implicated T cells in LC, albeit with contradictory findings. While certain studies have reported increased SARS-CoV-2-specific T cell responses in LC in comparison to non-LC cases [184,221], a rapid decay of subsets of SARS-CoV-2-specific CD8^+^ T cells was observed in the context of LC in another study [187]. In addition, the kinetics of antigen-specific T cell responses varied in LC, with individuals presenting similar antigen-specific T cell responses at the early and intermediate phases of convalescence and even elevated responses at a later time point [184]. In one study, a decreased proportion of CD8^+^ T cells expressing CD107a, a marker of degranulation, in response to the N peptide pool stimulation, and a faster decline in the frequency of N-specific interferon-γ-producing CD8^+^ T cells were observed in patients with LC after 4 months of COVID-19 onset [187].

### 6.5. Biomarkers Reflecting Reactivation of Latent Viruses

Herpesviruses are ubiquitous and may establish lifelong latency following primary infection. The inability to maintain latency with short-term or long-term implications may be caused by other infections. In a recent meta-analysis, the prevalence of active herpesviruses infections in COVID-19 patients was 41% for EBV, 3% for HHV-6, 28% for HSV, 25% for CMV, 22% for HSV1 and 18% for VZV, while severe COVID-19 is associated with a 6-fold higher chance for active EBV infection [235]. Interestingly, EBV reactivation in the acute phase of COVID-19 infection may be associated with severe COVID-19 [236].

Reactivated herpesviruses, particularly EBV and HHV-6, were documented in patients with LC [174,188,189,190,191]. Regarding EBV reactivation, EBV viremia or increased titers of anti-EBV antibodies (IgM/IgG against viral capsid antigen/VCA and IgG against EBV-encoded nuclear antigen/EBNA) may be early predictive biomarkers of LC. Reactivation may occur soon after or concurrently with COVID-19 infection [174,188,189,190,191,235]. One study demonstrated that early EBV viremia may be associated with fatigue and respiratory symptoms [190], while in two studies increased antibody titers against EBV were associated with fatigue, cognitive dysfunction [188], headache, psycho-neurological disorders, respiratory symptoms and myalgia, and increased liver enzymes, CRP and D-dimer [189]. The reactivation of EBV infections has also been found amid patients with ME/CFS, suggesting that EBV is an important factor for the development of the disease [90,237]. Interestingly, an altered and chronically aroused antiviral profile against latent viruses (EBV, HHV6 and human endogenous retrovirus K), particularly IgG against EBV-encoded nuclear antigen-1 (EBNA1), was noted in patients with ME/CFS after mild/asymptomatic COVID-19 [238].

### 6.6. Biomarkers Reflecting Autoimmunity

Infections may trigger antibody polyreactivity and autoimmunity, which are generally considered detrimental [239]. COVID-19 encompasses a broad spectrum of clinical phenotypes that exhibit exaggerated and misdirected host immune responses [240]. Patients with COVID-19 show marked elevations in autoantibody reactivities as compared to uninfected people, with an increased prevalence of autoantibodies against immunomodulatory proteins (e.g., cytokines, chemokines, complement components and cell-surface proteins) that may alter immune function and impede the control of the virus [241]. Autoantibodies, particularly those that neutralize type I IFNs, have been documented to be related with immune dysregulation and COVID-19 severity and death [242,243,244,245] and have been proposed to be associated with LC.

To date, contradictory results have been reported for autoantibodies as a major component in the pathogenesis of LC. In particular, there are studies that did not report a specific profile of autoantibodies, including anti-IFN and anti-IFN-α2 autoantibodies or antinuclear (ANA) antibodies [196,246], that could differentiate patients with LC from recovered patients or controls [174,193,196,246]. On the contrary, other studies have reported a plethora of autoantibodies in LC, including elevated ANA antibodies (e.g., anti-U1-snRNP and anti-SS-B/La), antineuronal antibodies, anti-IFN antibodies, autoantibodies against ACE2, the angiotensin II AT_1_ receptor, the angiotensin 1–7 MAS receptor, β_2_-the adrenoreceptor and the muscarinic M2 receptor [146,190,194,195,197,247]. Interestingly, in one cohort study, the presence of antibodies against specific chemokines (CCL21, CXCL13 and CXCL16) was negatively associated with the development of LC at 1-year post-infection [192]. It is possible that autoantibodies to certain chemokines may positively impact the long-term outcome of COVID-19 by antagonizing or regulating the activation, recruitment and retention of activated T and B cells in diverse anatomic sites [192]. Finally, in some studies, specific autoantibodies were associated with clinical phenotypes of LC. ANAs were associated with persistent symptoms of fatigue and dyspnea [194], antineuronal antibodies in CSF and serum with cognitive impairment [195], and anti-IFN-α2 or anti-IFN-λ autoantibodies with respiratory symptoms of LC [190,197].

### 6.7. Endothelial or Vascular Biomarkers

Recent data have shown that endothelial inflammation and damage in COVID-19 could lead to long-term implications for vascular function [199,248]. Endotheliopathy is also observed in LC and is associated with the disease [94]. Furthermore, increased inflammatory mediators in LC may lead to the upregulation of cell adhesion molecules and angiogenesis factors and the shedding of the protective glycocalyx matrix of the capillary endothelium with subsequent significant alterations in microvascular resistance and hemodynamics [94,249]. The adhesion of hyperactivated neutrophils to capillaries in the brain, lungs, heart and other tissues or organs may further be implicated in the pathogenesis of LC [94,127]. In a recent observational study, elevated endothelin-1, a biomarker that mediates vasoconstriction, and decreased angiopoietin-2, a biomarker of vascular angiogenesis, were reported in patients with LC and fatigue [199]. Interestingly, decreased angiopoietin-2 was found exclusively in LC and could be a differentiation biomarker between LC and ME/CFS [199]. In another study, angiopoietin-1 and P-selectin, a protein synthesized by activated thrombocytes and endothelial cells that acts as a cell adhesion molecule, presented excellent sensitivity and specificity for predicting LC status among 16 blood biomarkers of vascular transformation [198]. However, a larger cross-sectional study did not find significant differences in vascular biomarkers between patients recovered from COVID-19 one year after hospital discharge and controls [200].

### 6.8. Biomarkers of Coagulation and Fibrinolysis

Endothelial dysfunction and hypercoagulability may present in some patients approximately 1 year after recovery from COVID-19 [250]. Interestingly, some patients with LC have a genetic predisposition for thrombophilia based on the evaluation of related genetic polymorphisms [250]. Individuals with LC may present with anomalous fibrin amyloid microclots that block capillaries and cause tissue hypoxia, dysfunction in oxygen exchange and tissue damage upon restoration of oxygen supply (ischemia–reperfusion injury) [94,251]. These microclots, which are resistant to fibrinolysis, may trigger oxidative stress and the secretion of proinflammatory cytokines, establishing a vicious cycle that may be implicated in the plethora of clinical manifestations observed in LC [94,204,251]. Several studies have shown that microclots were identified in the plasma of patients with LC, carrying altered levels of coagulation and fibrinolysis proteins, which are involved in endothelial injury, platelet activation, coagulation and fibrinolysis, such as increased von Willebrand factor (vWF), platelet factor 4, fibrinogen chains α and β, factor XIII, plasminogen and antiplasmin (α2AP), and decreased plasma kallikrein [203,204]. Numerous proinflammatory molecules, such as SAA, antibodies and complement components trapped inside microclots, were also detected by proteomics in LC [124,203,204,252]. Interestingly, ME/CFS is also characterized by substantial and measurable alterations in platelet hyperactivation, coagulability and fibrinoid microclot formation; nevertheless, the load of microclots is greater in LC [132].

A meta-analysis of 23 prospective and retrospective studies with 18 meta-analyzed biomarkers has shown that elevated D-dimer presents a diagnostic utility in LC, albeit of small effect, and is present in some patients with organ abnormalities and in patients with a duration of symptoms of more than 6 months [172]. Concentrations of D-dimer have also been associated with severe COVID-19 infection [253]. Interestingly, abnormal D-dimer levels were found in children with LC and more symptoms at 12-weeks post-infection compared to children that had fully recovered at 8–12 weeks [202]. Collectively, these findings underscore the contribution of endotheliopathy, coagulopathy and failed fibrinolysis in the pathogenesis of LC.

### 6.9. Hormonal and Metabolic Biomarkers

Endocrine dysfunction may also be implicated in LC pathogenesis. Two longitudinal multiomics studies have reported persistent low levels of cortisol as a robust distinctive characteristic between patients with LC, convalescent individuals and healthy controls [174,190]. In the yet unpublished, exploratory, cross-sectional and longitudinal study of 220 individuals, the plasma cortisol in patients with LC was half of that in age-, sex- and BMI-matched controls at 400-days post-infection, while hypocortisolemia was the most significant predictor for LC occurrence based on machine learning tools [174]. Moreover, a recent multiomics, longitudinal study has shown that low levels of plasma cortisol in conjunction with the presence of anti-IFN autoantibodies were found in patients with LC and respiratory symptoms 3 months later [190]. Intriguingly, low cortisol secretion by the adrenal glands was not compensated by an increase in adrenocorticotropic hormone (ACTH), suggesting a dysfunction in the hypothalamus–pituitary–adrenal (HPA) axis. This dysfunction is compatible with clinical manifestations present in primary adrenal insufficiency (Addison’s disease), such as fatigue, brain fog, muscle weakness, nausea, abdominal pain, body hair loss, anxiety or depression, etc. [254]. Interestingly, hypocortisolemia with the hyporesponsiveness of the HPA axis and chronic fatigue are observed in patients with ME/CFS, chronic stress disorders and fibromyalgia and some post-viral infection syndromes, such as post-SARS and post-MERS [83,135,255,256,257]. Low cortisol has also been documented in acute COVID-19 [258]. Notably, the triad of hypocortisolemia, reactivation of EBV and T -cell exhaustion has also been reported in ME/CSF, which accounts for a significant percentage of LC patients [259,260].

Regarding other hormonal and metabolic alterations, low cortisol was associated with dysosmia/dysgeusia in a retrospective analysis of 186 patients with LC but without convalescent or control patients. Low serum growth hormone and increased serum FT4 were linked to general fatigue, while higher thyrotropin (TSH) and lower FT4/TSH were found in the initially severe LC cases [207]. In another retrospective cohort study, patients with LC presented with increased new-onset IR, while increased IR was significantly linked to depressive symptoms [137]. Interestingly, lower adiponectin, which is associated with obesity, metabolic syndrome, IR and cancer [261,262,263,264,265,266,267,268,269,270,271], was detected more often in digested microclots of LC patients compared to the control group [203]. Finally, there was a 42% relative reduction in the incidence of LC in a metformin group compared to its blinded control in a randomized trial of 1125 adults in the US aged 30 to 85 who were overweight or obese [272].

A growing body of evidence based on meta-analyses, observational prospective and retrospective studies, and interventional studies has shown associations between hypovitaminosis D and the occurrence of acute severe COVID-19, including an elevated risk of admission at the ICU, length of stay at the ICU, need for mechanical ventilation and mortality [75,273,274]. Vitamin D presents well-known extra-skeletal properties, including the regulation of both innate and adaptive immunity as well as enhanced activity against viruses, bacteria and fungi through the induction of cathelicidin and defensins [275,276,277]. Moreover, the beneficial effects of vitamin D have been consistently acknowledged in cardiometabolic, respiratory, neurocognitive and musculoskeletal health [278,279,280,281,282,283]. To date, only a few studies have explored the association between vitamin D and the occurrence of LC, reporting conflicting findings [205,206,208,284,285]. In a cross-sectional controlled study, a lower 25OHD level was an independent predictor of LC occurrence and characterized patients with neurocognitive symptoms at follow-up [205]; however, other studies did not document any association between vitamin D levels and post-COVID symptoms [206,208] or the use of vitamin D for the management of persistent or new symptoms post-COVID [284]. Therefore, larger prospective and longitudinal studies as well as randomized controlled trials with vitamin D supplementation are required to elucidate the role of vitamin D in LC.

### 6.10. Various Proteins as Biomarkers

In a prospective longitudinal cohort study of 2320 participants discharged from hospital, plasma proteome data have shown that 13 inflammatory proteins were associated with the severe group of LC [92]. The highest protein concentration was trefoil factor 2 (TFF2), a protein released with mucin from mucosal epithelium comprising lung and gastric mucosa, suggesting persistent mucosal epithelial abnormalities and inflammatory cell activation. Increased IL-6 and CD70 were associated with cognitive impairment, highlighting the role of CD70 in neuroinflammation in the CNS. Furthermore, higher serum agrin, which has been detected in older adults with sarcopenia, was associated with physical impairment [92]. In another longitudinal proteomic case-control study of 156 healthcare workers, a plasma proteomic signature associated with lipid, atherosclerosis and cholesterol metabolism pathways, complement and coagulation cascades, autophagy and lysosomal function at the time of seroconversion had the potential to predict patients who are more likely to suffer from LC [209]. Finally, another proteomic study found evidence of a failed fibrinolytic system in LC associated with the entrapment of many proinflammatory proteins, which may be important for patients with pre-existing comorbidities, including cardiovascular disease and type 2 DM [203].

### 6.11. Metabolites as Biomarkers

Metabolomics could be a powerful tool for diagnostic, prognostic and drug intervention analysis in COVID-19 as well as in LC [286]. Furthermore, metabolomics may provide non-invasive determinations of plasma and tissue mitochondrial-related metabolites. Although there is no characteristic metabolite signature associated with LC, some studies have shown that LC, even in the absence of severe acute COVID-19, may present the following: (1) an altered fatty acid metabolism and dysfunctional mitochondria-dependent lipid catabolism consistent with previously reported mitochondrial dysfunction during exercise and decreased fatty acid oxidation capacity of mitochondria [150]; (2) an impaired pyruvate/lactate metabolism characterized by decreased levels of carboxylic acids [150,211]; (3) an accumulation of free and carnitine-conjugated fatty acids associated with erythrocyte dysfunction, which may impair oxygen delivery to tissues [150]; (4) an activation of the kynurenine pathway, where specifically the elevation of the metabolites quinolinic acid, 3-hydroxyanthranilic acid and kynurenine is linked to cognitive impairment in LC [212]; (5) a dysregulation in the sphingolipid metabolism that could be associated with fatigue and muscular pain in LC [210]. Overall, based on metabolomics studies, mitochondrial dysfunction, impaired energy metabolism, altered lipid metabolism and redox state imbalance may characterize LC. Future larger prospective and longitudinal studies are required to explore mitochondrial biology, oxygen and lactate kinetics. Finally, interventions targeting the promotion of mitochondrial biogenesis and the restoration of mitochondrial function are much awaited.

### 6.12. Microbiota Alterations in Long COVID

Gut dysbiosis may contribute to the development of several metabolic, autoimmune and inflammatory disorders, including ME/CFS [267,287,288,289]. Several lines of evidence have shown the replication of SARS-CoV-2 in human enterocytes and its detection in stool samples as well as an altered intestinal microbiota composition in patients with COVID-19, which correlate with disease severity and inflammatory biomarkers [290]. Gut dysbiosis could persist after disease resolution and be implicated in LC [291]. Indeed, based on some prospective follow-up studies in recovered COVID-19 patients and controls, gut microbiota dysbiosis may be present one year after discharge in recovered COVID-19 patients, being predictive of LC occurrence [213,214].

Patients with LC may present the following g: (1) reduced bacterial diversities; (2) a lower relative abundance of short-chain fatty acid (SCFA)-producing beneficial symbionts, such as *Eubacterium hallii group*, *Subdoligranulum*, *Ruminococcus*, *Dorea*, *Coprococcus* and *Eubacterium_ventriosum* [213,214]; (3) lower levels of butyrate-producing bacteria that are associated with LC at 6 months [214]; (4) an increased abundance of *Ruminococcus gnavus* and *Bacteroides vulgatus* and decreased abundance of *Faecalibacterium prausnitzii* [214]. Interestingly, microbiota transfer from individuals with LC provoked a decline in brain cognitive properties and dysfunctional lung defense in mice, which was partially prevented by the administration of the commensal probiotic *Bifidobacterium longum* [292]. Probiotics, prebiotics and synbiotics, which have shown beneficial metabolic and immunologic properties [277,288,293,294], could be used as adjunct therapeutic tools in LC in the future. Finally, although there are no data on mycobiota alterations in LC, a study has found increased levels of fungal translocation from the gut and/or the lung epithelia, determined by β-glucan, a fungal cell wall polysaccharide, in the plasma of individuals of LC, suggesting the important role of fungal translocation in chronic immune activation due to gut barrier permeability [295].

Overall, all these findings suggest that an individual’s intestinal microbiome configuration at the time of SARS-CoV-2 infection may alter the susceptibility to long-term complications of COVID-19. Nonetheless, these alterations may represent reactive changes to LC; therefore, future research including prospective longitudinal studies with a multiomics approach of non-hospitalized patients followed from the time of infection until the development of persistent symptoms is required to delineate the exact contribution of intestinal dysbiosis to LC.

### 6.13. Cerebrospinal Fluid Biomarkers

Cerebrospinal fluid (CSF) analysis is a diagnostic mainstay in neuroinflammatory disorders. Nevertheless, limited information on CSF data in patients with LC is currently available [142]. In a systematic review, non-specific inflammatory CSF abnormalities were frequently found in patients with COVID-19 and neurological symptoms; however, the elevation of neurodegeneration biomarkers could suggest that neuronal damage may present unknown long-term implications [296]. In a retrospective multicenter analysis of 150 lumbar punctures in 127 patients with PCR-proven COVID-19 and neurological involvement, the CSF profile has shown markers of blood–brain barrier (BBB) disruption in the absence of intrathecal inflammation, which is compatible with cerebrospinal endotheliopathy [148]. Hyperalbuminorrachia and elevated levels of the astroglial protein S100B, suggestive of BBB dysfunction, were found in a small but extensive longitudinal study of COVID-19 patients with neurological manifestations [297]. This BBB dysfunction as well as the increased levels of cytokines and chemokines may be implicated in both acute neurologic symptoms as well as in LC. Another study with a small sample size in patients with neuro-COVID has shown an increase in CSF biomarkers of neuroinflammation and neurofilament light chain, indicative of neuronal damage [298]. In a cross-sectional and long-term longitudinal study with a multiomics approach, the prevailing signs of severe neuro-COVID were the dysfunction of the blood–brain barrier, increased microglia activation biomarkers and a polyclonal B cell response targeting self-antigens and non-self-antigens [142]. Patients with neuro-COVID presented with decreased regional brain volumes related to specific CSF markers; nonetheless, COVID-19 patients with a cytokine storm did not show an inflammatory CSF profile [142].

LC is more prevalent in patients with severe neuro-COVID and is linked to specific plasma and CSF biomarkers. In a long-term follow-up study, low levels of the anti-inflammatory protein TRANCE (RANKL) in conjunction with increased levels of the proinflammatory protein TNFRSF9 and IFN-γ levels were the best single protein predictors within CSF for LC [142]. Furthermore, the upregulation of plasma CLM-6, monocyte chemotactic protein (MCP)-3 and ST1A1, which are involved in macrophage and monocyte activation and infiltration in the central nervous system (CNS) and in autoimmune encephalitis, may predict LC [142,299]. After mild COVID-19, a sustained inflammatory response may not be a major component in the pathogenesis of LC with neurological symptoms based on CSF findings in subjects with cognitive impairment developing one to six months after recovery [215]. In this small case-control study within a prospective study evaluating recovery from COVID-19, although the majority of participants presented normal values in CSF for many parameters (white blood cells, glucose, CSF/serum albumin ratio, IgG index, etc.), a higher proportion of abnormal CSF findings and an abnormal oligoclonal banding pattern were detected in 77% and 69% of patients with LC and cognitive impairment versus 0% of controls, underscoring a potential abnormal autoimmune response [215]. Finally, in a prospective case-control study of 60 hospitalized neuro-COVID patients, only levels of protein 14-3-3 and neurofilament light chain (NfL) in the CSF, which are biomarkers of neuroaxonal damage, were significantly associated with a degree of neurologic disability in daily activities at 18 months follow-up [216].

Overall, all these biomarkers are associated with neuropathologic mechanisms, including peripherally induced cytokine alterations, followed by blood–brain barrier dysfunction with intrusive polyreactive autoantibodies, endothelial dysfunction and coagulopathy, increased reactivity of microglia, neuroinflammation and neuronal damage [114].

## 7. Biomarkers Classifying Clinical Manifestations in Long COVID

**Figure 2:**
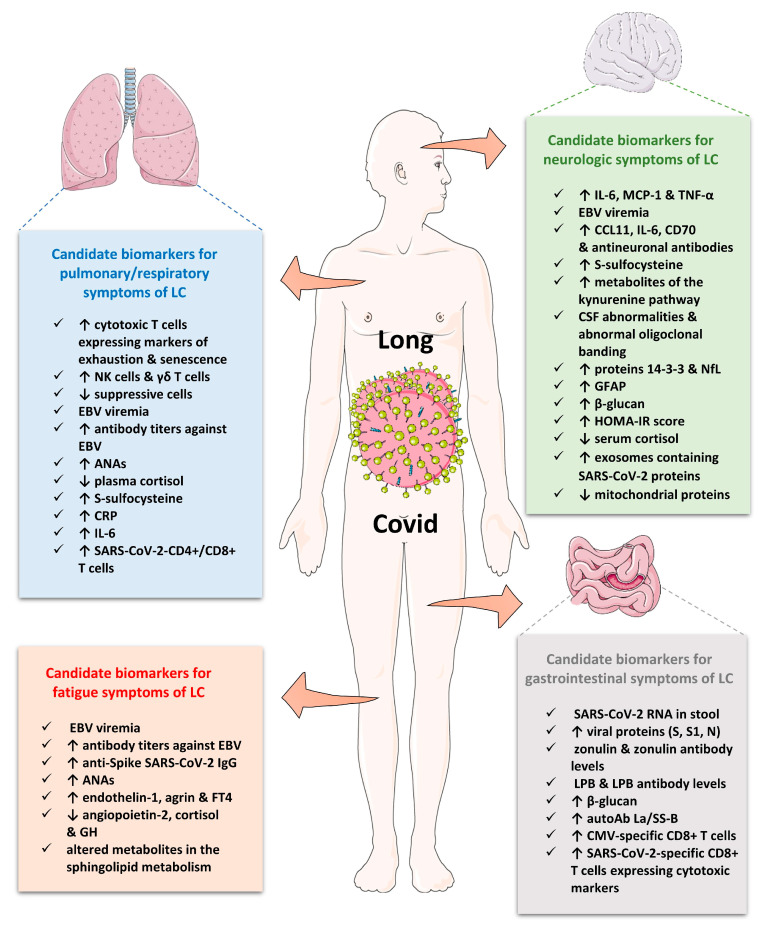
Candidate biomarkers classifying clinical manifestations in long COVID (LC). Abbreviations: ↑, increased; ↓, decreased; ANAs, antinuclear antibodies; CMV, Cytomegalovirus; CRP, C-reactive protein; CSF, cerebrospinal fluid; EBV, Epstein–Barr Virus; GFAP, glial fibrillary acidic protein; GH, growth hormone; HOMA-IR, Homeostatic Model Assessment for Insulin Resistance; IFN, interferon; IL, interleukin; LBP, Lipopolysaccharide Binding Protein; MCP, monocyte chemotactic protein; NfL, neurofilament light chain protein; NK, natural killer; SARS-CoV-2, Severe Acute Respiratory Syndrome Coronavirus 2; TNF, tumor necrosis factor (all images are originated from the free medical site http://smart.servier.com/ (accessed on 15 May 2023) by Servier licensed under a Creative Commons Attribution 3.0 Unported License).

LC represents a heterogeneous complex multisystem disorder with different clinical manifestations; hence, it would be ideal to categorize it for diagnostic, prognostic and therapeutic purposes. Notwithstanding that the majority of biomarkers analyzed in this review concern the totality of LC, candidate-specific biomarkers for major distinctive clinical features of LC are classified into the following groups: general symptoms including fatigue and muscle pain; respiratory symptoms; neurologic symptoms; and gastrointestinal symptoms. Figure 2 depicts the main candidate biomarkers for symptom-distinctive features of LC.

### 7.1. Candidate Biomarkers for General Symptoms and Fatigue

Overall, altered levels of D-dimer, LDH and lymphocytes were characteristic of patients with organ abnormalities evaluated by imaging and functional studies, whilst increased levels of IL-6, D-dimer and CRP were observed in some patients with a persistence of symptoms (more than 6 months) [25,92,172,178,204,220,221]. Interestingly, D-dimer was increased in children with LC and persistent symptoms [202]. In some patients, increased concentrations of IL-6 and CRP are informative of the early stage of LC [173,222]. Symptoms of fatigue and physical impairment are associated with early EBV viremia; increased antibody titers against EBV, antispike SARS-CoV-2 IgG at 2 months after infection and ANAs; higher serum endothelin-1, agrin and FT4; lower angiopoietin-2, cortisol and growth hormone; and altered metabolites in the sphingolipid metabolism [92,174,186,188,190,194,199,207,210]. More importantly, decreased angiopoietin-2 was reported exclusively in LC, differentiating LC from ME/CFS [199].

### 7.2. Candidate Biomarkers for Neurological Symptoms

Neurological symptoms are common in LC [20] and are associated with increased levels of proinflammatory cytokines IL-6, MCP-1 and TNF-α in certain patients [300]. Cognitive dysfunction was associated with the reactivation of EBV; increased CCL11, IL-6, CD70 and antineuronal antibodies; elevated concentrations of S-sulfocysteine; and higher levels of metabolites of the kynurenine pathway (quinolinic acid, 3-hydroxyanthranilic acid and kynurenine) [137,188,195,212,225,226,295]. In CSF, cognitive impairment was associated with a greater frequency of CSF abnormalities and abnormal oligoclonal banding; a higher frequency of antineuronal antibodies; and increased levels of proteins 14-3-3 and NfL [195,215,216]. Elevated levels of cytoskeletal proteins NfL and glial fibrillary acidic protein (GFAP), which maintain the stability of neuronal axons and astrocytes, and β-glucan were linked to worse headaches and persistent neuropathic pain in patients with LC [300,301]. Depressive symptoms were linked to new-onset IR, as expressed by an increased Homeostatic Model Assessment for Insulin Resistance (HOMA-IR) score [137], while dysosmia/dysgeusia was associated with low serum cortisol [207]. Finally, elevated levels of exosomes containing SARS-CoV-2 proteins and lower levels of mitochondrial proteins may be promising biomarkers for neuropsychiatric symptoms [302]; however, more studies are required to delineate their significance in neurological LC.

### 7.3. Candidate Biomarkers for Respiratory Symptoms

Pulmonary lesions represent frequent sequelae of SARS-CoV-2 infection with a considerable proportion of COVID-19 survivors from previous variants presenting residual lung lesions, such as ground-glass opacity and fiber streak shadow [303]. A cough and dyspnea are the most frequent and persistent symptoms lasting more than 7 months in 20% and 40% of patients, respectively [304]. In a study with convalescent patients followed one year after discharge, patients with pulmonary sequelae presented a prominent immunologic profiling characterized by the activation of cytotoxic T cells expressing markers of exhaustion and senescence, NK cells and γδ T cells, as well as a deficit of suppressive cells [303]. In other patients, respiratory symptoms were associated with early EBV viremia and increased antibody titers against EBV [188,190]; elevated ANAs and anti-IFN-α2 or anti-IFN-λ autoantibodies [190,194,197]; low plasma cortisol [190]; and higher S-sulfocysteine [295]. Finally, another study has shown that patients with respiratory symptoms of LC exhibited higher levels of CRP, IL-6 and SARS-CoV-2-CD4^+^/CD8^+^ T cells secreting IFN-γ or TNF-α at 7-months post-infection [221].

### 7.4. Candidate Biomarkers for Gastrointestinal and Other Specific Symptoms

Promising biomarkers for gastrointestinal manifestations in LC and MIS-C include SARS-CoV-2 RNA in stool and increased circulating viral proteins (S, S1, N) and increased levels of zonulin, LPB, β-glucan, autoAb La/SS-B and CMV-specific CD8^+^ T cells as well as elevated SARS-CoV-2-specific CD8^+^ T cells expressing cytotoxic markers [25,180,183,190,295]. Finally, a prospective observational study one year after the hospital discharge of 263 COVID-19 survivors found that 26.2% had hair loss. Interestingly, lower levels of Growth Arrest-Specific 6 (Gas6) and its soluble receptor Axl (sAxl) were associated with a history of alopecia [305].

## 8. Limitations of Studies and Challenges

In this review, we have appraised and summarized biomarkers related to LC. Nevertheless, no specific biomarker and laboratory test or panel of biomarkers may differentiate LC from other disease entities with adequate certainty. Previous data have shown that many phenotypes and distinct subdiagnoses could exist under the umbrella of LC; therefore, sequelae of COVID-19 may certainly not be due to a unique pathogenetic pathway. Moreover, due to the heterogeneous and multifaceted pathogenesis of LC, the diagnostic and predictive performance of the majority of biomarkers is modest. Furthermore, considering that patients fulfilling the definition of LC may or may not exhibit objective organ damage [67], candidate biomarkers could ideally aid to pinpoint the subset of patients with target organ involvement. Still, to date evidence in this direction remains inconclusive, with no biomarker exhibiting sufficient accuracy to distinguish specific patient categories with target organ damage, despite its high prevalence among those with LC [67].

There are many methodological limitations in the studies evaluating laboratory findings and biomarkers for the prediction, diagnosis and prognosis of LC. In the beginning, there was an absence of a consensus disease definition for LC, which led to the misclassification of subjects. Misclassification could arise when it is difficult to differentiate long-term symptoms potentially attributed to LC from symptoms due to other conditions both related or unrelated to LC. Another significant shortcoming was the reliance on self-reported information regarding LC status, which could lead to selection bias. The timing of symptom onset is also subject to recall bias, particularly when information is collected many months after acute COVID. More importantly, some studies focused on hospitalized cohorts, which are not representative of the general population. Other issues include the false negative rate of PCR tests, which could classify participants as controls, as well as the limited utility of antibody titers to determine previous SARS-CoV-2 infection [11]. On the contrary, some studies did not stipulate a laboratory-confirmed diagnosis of COVID-19 and focused on the most common symptoms and different time periods with great variability [306].

Most studies were underpowered due to small sample sizes, particularly for control groups, and included convenient cohorts with participants who were not lost in follow-up. Many studies had a retrospective design where most of the biomarkers were determined after the onset of LC symptoms; hence, altered biomarkers could be an epiphenomenon of LC. Finally, the majority of studies were conducted in the early phase of the pandemic, examining a limited set of biomarkers; therefore, it is not clear how these biomarkers are useful for LC due to Omicron subvariants.

## 9. Therapeutic Perspectives and Challenges

Although most therapeutic approaches put to test against LC have targeted symptom alleviation [307], a comprehensive evaluation of symptoms and clinical signs of affected patients is imperative, in order to timely recognize “red flag” features pointing towards underlying objective systemic disorders that are likely to have a decisive impact on patient prognosis. Likewise, meticulous laboratory, radiological and other functional investigations should be reserved for patients exhibiting such features and not necessarily be broadly offered as a screening process in all cases of ongoing symptoms following acute infection. Overall, in order to optimize patient access to adequate care and reduce the burden on secondary and tertiary hospitals, evaluation and initial management ought to be commissioned to primary care units, after adequate information and the education of primary care physicians. Ideally, primary patient care should be based on uniform evaluation algorithms and standardized procedures. Regarding patients exhibiting “red flag” symptoms or manifestations of debilitating magnitude, a further referral to specialist care should be carried out through interdisciplinary ambulatory COVID clinics, offering facilitated access to specialized personnel and investigations, essentially acting as a link between primary and hospital care [308].

Not surprisingly, for a constellation of symptoms and disorders as broad as those described under the umbrella term “LC”, no single therapeutic intervention with a proven benefit for all affected patients exists. However, there exist certain candidate treatments that may be potentially effective for specific patient groups or symptom clusters. Among those with chronic fatigue, interventions with a demonstrated benefit in ME/CFS may prove useful, most prominently lifestyle management (pacing, optimization of energy management, regular rests, etc.) or even pharmacologic interventions such as naltrexone [11,308]. Similar to the management of other causes of dysautonomia with postural hypotension, these findings in the context of LC may benefit from a high salt and fluid intake, beta-blockers, low-dose fludrocortisone and desmopressin, among others, depending on clinical presentation [309].

Among agents approved for the treatment of SARS-CoV-2 infection, the combination of nirmatrelvir with ritonavir (marketed under the name Paxlovid) has shown potential effectiveness in the mitigation of LC symptoms. Geng et al. reported a case of a patient with ongoing symptoms 6 months following a breakthrough SARS-CoV-2 infection, which were alleviated after a 5-day Paxlovid course [310]. Furthermore, those who received Paxlovid for 5 days (n = 35,717) in a recent study among 281,793 patients who had at least 1 risk factor for progression to severe COVID-19 had a 26% lower risk of LC compared with those receiving no antiviral or monoclonal antibody treatment [311]. Nevertheless, other cohort studies have failed to demonstrate corresponding benefits on long-term COVID sequalae via Paxlovid treatment [312,313].

Despite the lack of specific therapeutic agents to tackle LC, the repurposing of drugs marketed for other indications may show promise towards that end. Metformin, a first-line drug for the treatment of DM type 2 with documented in vitro [314,315] and in vivo [315] activity against SARS-CoV-2, showed effectiveness for LC prevention in a recent randomized, placebo-controlled clinical trial [272]. Using a parallel-group design, 1125 ambulatory individuals who were overweight or obese and had a documented SARS-CoV-2 infection within three days prior to enrollment were randomized between rapidly titrated metformin treatment and a placebo and additionally between ivermectin and/or fluvoxamine treatment vs. placebo. The duration of all study interventions was 14 days. The diagnosis of LC 300 days following randomization was a prespecified study outcome and occurred at a 42% lower rate among those that received metformin vs. placebo. No additional effects were noted for ivermectin or fluvoxamine [272]. An important implication of these findings is the need for interventions as early as at first diagnosis of acute infection for LC prevention. Although the reported results are definitely promising, more studies are needed in order to generalize these findings in a population with additional risk factors for LC other than being overweight/obese.

AXA1125 is a novel orally administered endogenous metabolic modulator structurally consisting of five amino acids, which improves mitochondrial efficiency and promotes beta oxidation [316]. In a recent randomized controlled trial, the oral administration of AXA1125 twice daily for four weeks to patients with fatigue-predominant LC resulted in greater symptomatic improvement vs. a placebo (assessed by the 28 Chalder Fatigue Questionnaire [CFQ-11] fatigue score). There were no improvements in skeletal muscle mitochondrial function assessed by magnetic spectroscopy. Although these findings are definitely promising, the relatively short follow-up duration of this study should be taken into account [317].

To date, more than 36 randomized trials have been registered in ClinicalTrials.gov [318], which include a mixture of dietary and herbal supplements, vitamins, cell-based treatments, anti-inflammatory agents, steroids, anticoagulants, antidepressants, lifestyle intervention, etc. All these heterogeneous trials are not generally focused on a cluster of LC, and they are small in size, which does not correspond to the millions of prevalent LC cases. Larger multicenter high-quality and methodologically robust randomized placebo-controlled trials conducted with a standardization of outcomes are needed to confirm any benefit from treatments found in smaller trials [319].

## 10. Concluding Remarks-Quo Vadis?

The constellation of symptoms and disorders in the spectrum of LC, though seldom life-threatening, have a significant negative impact on individual functional status and quality of life. Given its high incidence and prevalence following acute infection, LC is a condition with a considerable societal and health care impact. With rates of direct morbidity and mortality from acute infection subsiding due to broad vaccination- and infection-related immunity as well as less virulent SARS-CoV-2 strains, the relative impact of long-term sequelae associated with SARS-CoV-2 infection is gaining importance.

There is an imperative need to determine not only the features of those at high risk for LC but also the specific characteristics within patient groups most likely to benefit from therapeutic interventions. Since clinical risk factors themselves do not suffice for this task, the focus has been set on the recognition of sensitive and reliable diagnostic biomarkers. Unfortunately, this effort has yielded few robust results to date, due to being plagued, among other things, by methodological issues pertinent to the available, mostly retrospective, epidemiological studies. Future attempts should be focused on evaluating ideally readily available serum, radiological or other biomarkers, which will additionally aid to shed more light on the underlying mechanisms that drive LC development. In turn, these should be put to test in the frame of randomized clinical trials of candidate therapeutic interventions, in order to promote precision medicine in the management of affected patients.

## Figures and Tables

**Table 1:** Overview of definitions for long COVID.

Source	Used Terms	Definition
World Health Organization (WHO) [12]	Post-COVID condition or long COVID	The continuation or development of new symptoms 3 months after the initial SARS-CoV-2 infection lasting for at least 2 months with no other explanation.
National Institute for Clinical Excellence (NICE) [13]	Long COVID, ongoing symptomatic COVID-19, post-COVID	Long COVID: Signs and symptoms that continue or develop after acute COVID-19. It includes both ongoing symptomatic COVID-19 and post-COVID. Ongoing symptomatic COVID-19: signs and symptoms of COVID-19 lasting from 4 weeks up to 12 weeks. Post-COVID: Signs and symptoms that develop during or after COVID-19, continue for more than 12 weeks and are not explained by an alternative diagnosis. This diagnosis may be considered before 12 weeks while the possibility of an alternative underlying disease is also being assessed.
Centers for Disease Control and Prevention [14]	Long COVID or post-COVID conditions	Signs, symptoms and conditions that continue or develop (at least 4 weeks) after initial COVID-19 infection.
Robert Koch Institute [15]	Long COVID, post-COVID condition or post-COVID syndrome	Longer-term health impairments following a SARS-CoV-2 infection that are present beyond the acute phase of the sickness phase of four weeks. Post-COVID condition/post-COVID syndrome: the presence of symptoms for at least 12 weeks after the acute infection or symptoms that appear anew after this period and cannot be explained otherwise.
Government of Canada [16]	Post-COVID condition or long COVID	Symptoms of COVID-19 persisting for more than 12 weeks after the infection.

**Table 2:** Main risk factors and clinical and laboratory findings in acute severe COVID-19 and long COVID.

Main Characteristics	Acute Severe COVID-19	Long COVID
**Age**	Older age	↑ % of diagnoses between the ages of 36 and 50 years
**Gender**	More frequent in males	More frequent in females
**Predisposing factors and comorbidities**	- Older age, obesity, T2DM, CVD, asthma or chronic lung disease, sickle cell disease, immunocompromised patients, hematologic malignancies, chronic kidney disease, patients under immunosuppressive treatments - Racial and ethnic minority groups - People with low income - Unvaccinated individuals	- Obesity, T2DM, connective tissue disorders, allergic rhinitis, ADHD - More frequent in Black and Hispanic Americans - People with a low income - Poor rest in the early period after COVID-19 - ↑ risk after severe COVID-19 - Most cases involve non-hospitalized patients with a mild acute COVID-19
**Laboratory findings and biomarkers**	Lymphopenia, ↑ CRP, ↑ neutrophils, ↑ IL-6, ↑ IL-10, ↑ D-dimer, ↑ LDH, ↑ ferritin	The following factors are present in certain patients: ↑ IL-6, ↑ CRP, ↑ D-dimer, detectable SARS-CoV-2 RNA in stool or gut mucosa, biomarkers of EBV reactivation, anti-IFN-α2 or anti-IFN-λAbs, ↑ ET-1 and ↓ Ang-2, ↓ cortisol, metabolites of mitochondrial dysfunction, ↑ % of CSF abnormalities and ↑ biomarkers of neuronal damage
**CVD features**	Pulmonary embolism, deep vein thrombosis, AMI, heart failure	Chest pain, palpitations, myocarditis, cardiac impairment, POTS
**Neurologic features**	Headache, ischemic stroke, ataxia, seizures, anosmia, ageusia	Brain fog, fatigue, musculoskeletal pain, cognitive impairment, paresthesia, sleep disorder, dizziness, memory loss, dysautonomia, tinnitus
**Pulmonary features**	Cough, dyspnea, hypoxia, bilateral lung infiltrates	Cough, dyspnea, abnormal gas exchange, ground glass lung
**Gastrointestinal features**	Abdominal pain, nausea, vomiting, diarrhea, T1DM,↑ transaminases	Abdominal pain, nausea
**Endocrine abnormalities**	↑ T1DM, ↑ T2DM thyroiditis	Diabetes, hypocortisolism
**Dermatologic findings**	Skin rashes (maculopapular, chilblain-like, urticarial, vesicular, livedoid and petechial lesions), hair loss	Most commonly alopecia, various skin rashes
**Renal manifestations**	Acute Kidney Injury, Acute Tubular Necrosis	↓ eGFR
**Manifestations from the reproductive system**	Menstrual irregularities, ↑ urinary frequency and nocturia	Menstrual irregularities, ↑ premenstrual symptoms, erectile dysfunction, ↓ sperm count

Abbreviations: ↑, increased; ↓, decreased; ADHD, attention deficit hyperactivity disorder; AMI, acute myocardial infraction; Ang, angiopoietin; CRP, C-reactive protein; CVD, cardiovascular disease; eGFR, estimated Glomerular Filtration Rate; ET, endothelin; IFN, interferon; IL, interleukin; POTS, postural orthostatic tachycardia syndrome; T1DM, diabetes mellitus type 1; T2DM, diabetes mellitus type 2.

**Table 3:** General clinical, laboratory and imaging diagnostic approach.

General Laboratory Work-Up
Complete blood count
Erythrocyte sedimentation rate
General biochemical tests: glucose, HbA1c, urea, creatinine, electrolytes (sodium, potassium, calcium, phosphate)
Indices of hepatic function: AST, ALT, γ-GT, ALP
CPK, ferritin, LDH
Indices of cardiac function: troponin, BNP or NT-proBNP
Indices of thyroid function: TSH, fT4
Indices of coagulation and fibrinolysis: D-dimer, fibrinogen, PT, aPTT
Indices of inflammation: CRP, IL-6
Vitamins: 25-OHD, B12
Autoantibodies and complements: RF, anti-CCP, ANA, ENA, ACA, autoantibodies against central nervous system antigens, C_3_, C_4_
**Imaging and Other Function Tests**
High-resolution Chest Computed Tomography
Computed Tomography Pulmonary Angiogram (CTPA)
Pulmonary function tests (spirometry, diffusion capacity, lung volumes)
Pulse oxymetry
Six-minute walk test (6MWT)
Electrocardiogram
Cardiac ultrasound
Cardiac magnetic resonance
Cardiopulmonary exercise testing (CPET)
Brain magnetic resonance
**Questionnaires and Clinical Tests**
Tilt table test for POTS or 10-Minute NASA Lean Test for orthostatic intolerance
Dyspnea scales (mMRC, NYHA)
Questionnaire SBQ-LC
Questionnaires for anxiety: BAI, HAM-A, GAD-7
Questionnaires for depression: BDI, HAM-D, PHQ-2,-9
**Some Promising Tests**
Cortisol/four-point salivary cortisol test
IgG, IgA, IgM
Natural killer cell function tests
Panels for reactivated herpesviruses (EBV, CMV, VZV, HHV-6)
SARS-CoV-2 RNA in stool or gut mucosa
Endothelin-1 and Angiopoietin-2
Hyperpolarized gas magnetic resonance of the lungs

Abbreviations: ACA, anticardiolipin antibodies; ALP, alkaline phosphatase; ALT, alanine transaminase; ANA, antinuclear antibodies; anti-CCP, anticyclic citrullinated peptide antibodies; AST, aspartate aminotransferase; aPTT: activated partial thromboplastin time; BAI, Beck’s Anxiety Inventory; BDI, Beck Depression Inventory; BNP, B-type natriuretic peptide; CMV, Cytomegalovirus; CTPA, CT pulmonary angiogram; CRP, C-reactive protein; CPK, Creatine Phosphokinase; DLCO: diffusing capacity of carbon monoxide; EBV, Epstein–Barr Virus; ECG: electrocardiogram; ENA: antibodies against extractable nuclear antigen; fT4, free thyroxine; GAD-7, Generalized Anxiety Disorder scale; HAM-A, Hamilton Anxiety Scale; HAM-D, Hamilton Depression Scale; HHV-6, Human HerpesVirus-6; Ig, immunoglobulin; LDH, lactate dehydrogenase; NASA, National Aeronautics and Space Administration; NT-proBNP, N-terminal pro b-type natriuretic peptide; PHQ-2,9, Patient Health Questionnaire; POTS, postural orthostatic tachycardia syndrome; PT, prothrombin time; RF, rheumatoid factor; SBQ-LC, Symptom Burden Questionnaire for LC; TSH, thyroid-stimulating hormone; VZV, Varicella-Zoster Virus; γ-GT, gamma-Glutamyl Transferase; 25-OHD, 25-hydroxyvitamin D.

**Table 4:** Major studies depicting potential predictive, diagnostic and prognostic biomarkers in long COVID.

**Study, Year**	Study Design/Population	Main Findings	Utility
***Biomarkers of systemic inflammation***			
Yong, S.J. et al., 2023 [172]	Meta-analysis of 23 studies (14 prospective and 9 retrospective case control) with 18 meta-analyzed biomarkers	- ↑ CRP, D-dimer, LDH and leukocytes in LC patients than those without LC - ↑ lymphocytes and IL-6 in LC patients than those without LC - ↑ IL-8 in LC than healthy controls	- Diagnostic utility in LC; however, the effects are small; - ↑ D-dimer, LDH and lymphocytes in patients with organ abnormalities - ↑ IL-6 in patients with symptom persistence - ↑ LDH, leucocytes and NT-pro-BNP in patients with duration of symptoms < 6 months - ↑ D-dimer in patients with duration of symptoms ≥ 6 months
Yin, J.X. et al., 2022 [173]	Meta-analysis of 22 case-control observational studies	- ↑ IL-6 levels in LC patients than controls - ↑ IL-6 levels in LC patients than those with non-post acute sequelae of severe COVID-19 - ↑ IL-6 levels in patients with acute COVID-19 than patients with LC	- IL-6 may predict LC; - IL-6 may characterize an early stage of LC.
Lopez-Leon, S. et al., 2021 [20]	A total of 21 meta-analyses on the prevalence of long-term effects in LC, 15 studies, 47,910 patients (age: 17–87 years).	- ↑ CRP in 8% of patients (95% CI: 5–12) - ↑ ferritin in 8% of patients (95% CI: 4–14) - ↑ procalcitonin in 4% of patients (95% CI: 2–9) - ↑ IL-6 in 3% of patients (95% CI: 1–7)	Limited evidence for systemic inflammation after approximately 4 months post-viral-infection.
***Immune profiling in long COVID***			
Klein, J. et al., 2022 [174]	A total of 220 participants (101 LC, 41 CC, 41 HC and 37 HCW) at >400-days post-infection, cross-sectional study	LC is characterized by the following: ↑ non-classical monocytes; ↑ activated B cells; ↑ double-negative B cells; ↑ exhausted T cells; ↑ IL-4/IL-6 secreting CD4 T cells; ↓ conventional DC1; ↓ central memory CD4 T cells.	The integration of immune phenotyping data into machine learning models may identify distinguishing features in the classification of LC.
Haunhorst, S. et al., 2022 [175]	Scoping review of three studies examining Tregs in LC	- Galán M et al. [176] found a 2.5X ↑ Tregs in LC compared to subjects that recovered completely. - Utrero-Rico et al. [177] ↓ Tregs in LC compared to subjects that recovered completely - ↓ Tregs in LC compared to seronegative controls [178]	No firm conclusions can be drawn about Treg alterations in LC.
Phetsouphanh, C. et al., 2022 [96]	LC group (n = 31) and asymptomatic matched controls (MC) (n = 31) compared to individuals infected with common cold coronavirus (HCoV) (n = 25) and unexposed healthy donors (n = 13)	↑ activated innate immune cells, ↓ naive T and B cells ↑ expression of type I IFN (IFN-β) and type III IFN (IFN-λ1) that remained persistently high at 8 months after infection	
Patterson, B.K. et al., 2021 [178]	Case-control and longitudinal study, 121 cases with LC, 29 healthy controls, 26 cases with mild–moderate COVID-19, 48 cases with severe COVID-19	-↑ B cells in LC compared to HC - ↑ inflammatory monocytes CD14^+^, CD16^+^ and CCR5^+^ compared to HC - ↓CD4^+^ and CD8^+^ T cells expressing PD-1 (exhausted lymphocytes) and T-reg in LC compared to HC	- ↑ inflammatory monocytes and ↓ exhausted lymphocytes detected in patients who later developed LC (predictive biomarkers) - ↑ inflammatory monocytes and ↑ exhausted lymphocytes in the convalescence period of LC
***Biomarkers reflecting SARS-CoV-2 persistence***			
Swank, Z. et al., 2022 [179]	A total of 63 individuals previously infected with SARS-CoV-2, 37 of whom were diagnosed with LC	↑ circulating spike protein in LC cases but not in convalescent controls up to 12 months	SARS-CoV-2 viral reservoirs may persist in the body.
Natarajan, A. et al., 2022 [180]	A total of 113 individuals with mild to moderate COVID-19, longitudinal study	- SARS-CoV-2 RNA in the feces at 4 months after diagnosis (12.7%) - SARS-CoV-2 RNA in the feces at 7 months after diagnosis (3.8%)	GI symptoms (abdominal pain, nausea, vomiting) are related to fecal shedding of SARS-CoV-2 RNA.
Stein, S.R. et al., 2022 [98]	Autopsies on 44 patients who died with COVID-19	- Persistent SARS-CoV-2 RNA in multiple anatomic sites, including throughout the brain, as late as 230 days - A proportion of ≥50% of late cases had persistent RNA in the myocardium and lymph nodes from the head and neck and from the thorax, sciatic nerve, ocular tissue and in all sampled regions of the CNS, except the dura mater.	SARS-CoV-2 can cause systemic infection and persist in the body for months in certain patients.
Tejerina, F. et al., 2022 [181]	Cohort of 29 patients who reported persistent symptoms at least 4 w after COVID-19	- A total of 45% had positive plasma RT-PCR results -A total of 51% were positive in at least one RT-PCR sample (plasma, urine or stool)	Potential systemic viral persistence associated with persistent symptoms
Zollner, A. et al., 2022 [182]	Cohort of 46 patients with IBD 219 days (range, 94–257) after confirmed COVID-19	- SARS-CoV-2 RNA in the gut mucosa ∼7 months after mild acute COVID-19 in 69.5% - Nucleocapsid protein persisted in 52.2% of patients in gut epithelium and CD8^+^ T cells - No detectable viral antigens in stool	- Viral persistence markers associated with symptoms of LC
Yonker, L.M. et al., 2021 [183]	A total of 19 children with MIS-C, 26 with acute COVID-19 and 55 controls.	- A total of 89% of cases with MIS-C had GI symptoms versus 27% of children with acute COVID-19 - Detectable viral loads in the stool (1.5 × 102 to 2.5 × 107 RNA copies/mL) in the majority of cases - ↑ plasma zonulin and LBP compared to controls - ↑ SARS-CoV-2 spike, S1 and nucleocapsid antigens compared to controls	- Prolonged exposure to SARS-CoV-2 in the GI tract of children with MIS -Loss of mucosal barrier integrity - SARS-CoV-2 antigenemia may trigger the hyperinflammatory responses defining MIS-C
***Humoral and cellular response against SARS-CoV-2 in long COVID***			
Klein, J. et al., 2022 [174]	A total of 220 participants (101 LC, 41 CC, 41 HC and 37 HCW) at >400-days post-infection; cross-sectional study	↑ IgG against spike and S1 in LC compared to vaccination-matched controls	- Potential predictive biomarker of LC
Files, J.K. et al., 2021 [184]	A longitudinal study of 50 cases with COVID-19: - Total of 20 with persistent symptoms (30–208 days); - Total of 30 with symptom resolution within 20 days.	The following were found in patients with persistent symptoms: - ↑ IgG avidity to SARS-CoV-2 spike protein; - sustained T cell response magnitudes; - ↑ antigen-specific CD4^+^ T cell responses against spike (late phase); - ↑ antigen-specific CD8^+^ T cell populations.	↑ T cell response magnitude in individuals with prolonged symptoms
García-Abellán, J. et al., 2021 [101]	Prospective, longitudinal study of 146 hospitalized COVID-19 patients	↓ peak IgG against spike-associated with LC symptoms at 6 months	- Potential predictive biomarker of LC
Augustin, M. et al., 2021 [185]	A longitudinal prospective cohort study of 958 non-hospitalized patients with confirmed COVID-19	↓ baseline levels of IgG against S1 domain of the spike associated with long-term symptoms	- Potential predictive biomarker of LC
Blomberg, B. et al., 2021 [186]	A prospective cohort study of 312 patients (247 home-isolated and 65 hospitalized)	- ↑ IgG against spike protein and ↑ microneutralizing antibody titers detected after 2 months independently associated with both persistent fatigue and total number of symptoms at 6 months	- Increased antibody titers is a predictive biomarker of LC symptoms
Peluso, M.J. et al., 2021 [187]	A prospective cohort study of 70 individuals with PCR-confirmed COVID-19	- No significant differences in early and late (1 and 4 months) antibody levels (IgG against SARS-CoV-2 Spike, RBD and two preparations of the N protein) between cases with and without persistent symptoms - No differences in T cell responses at initial time point between cases with and without persistent symptoms - ↓ CD8^+^ T cells expressing CD107a against N peptide in cases with LC -↓ N-specific interferon-γ-producing CD8^+^ T cells in cases with LC	
***Biomarkers reflecting reactivation of latent viruses***			
Peluso, M.J. et al., 2023 [188]	A cohort of 280 adults with prior COVID-19	- ↑ early antigen-diffuse EBV IgG positivity associated with fatigue at 4 m - ↑ IgG against EBNA associated with fatigue and neurocognitive dysfunction, at 4 m after infection	Reactivation of EBV is associated with fatigue and cognitive dysfunction in LC
Klein, J. et al., 2022 [174]	A total of 220 participants (101 LC, 41 CC, 41 HC and 37 HCW) at >400-days post-infection; cross-sectional study	↑ titers of anti-EBV antibodies, even though overall seroprevalence is not different from HC or CC	Altered humoral responses to distinct herpesviruses in LC as a predictive biomarker
Zubchenko, S. et al., 2022 [189]	A total of 88 patients with LC	- EBV reactivation in 42.6% - HHV6 reactivation in 25.0% - EBV plus HHV6 reactivation in 32.4%	Reactivation was associated with ↑ slight fever temperature, headache, psycho-neurological disorders, pulmonary abnormalities and myalgia, liver enzymes, CRP and D-dimer and ↓ cellular immune response
Su, Y. et al., 2022 [190]	A cohort of 309 COVID-19 patients from initial diagnosis to convalescence (2–3 months later), 457 HC	- EBV viremia at T1 was associated with LC (fatigue and respiratory symptoms) at 3 m post-infection.	- EBV viremia may be a predictive biomarker of LC.
Gold, J.E. et al., 2021 [191]	A cohort of randomly 185 surveyed COVID-19 patients, 56 developed LC	- ↑ EBV early antigen-diffuse IgG or ↑EBV viral capsid antigen (VCA) IgM in 66.7% of LC versus 10% of controls	- EBV reactivation may be a predictive biomarker of LC.
***Biomarkers reflecting autoimmunity***			
Muri, J. et al., 2023 [192]	A cohort of 71 COVID-19 convalescent patients at month 6 (on average) after disease onset, 23 HC	- IgG antibodies against chemokines CCL21, CXCL13 and CXCL16 at month 6 distinguished LC from no LC groups. - Levels of CCL21, CXCL13 and CXCL16 predicted the absence of persistent symptoms with 77.8% accuracy.	- Ab against specific chemokines were associated with a favorable disease outcome and negatively correlated with the development of LC at 1-year post-infection.
Bodansky, A. et al., 2023 [193]	A cohort of 121 individuals with LC, 64 with prior COVID-19 and full recovery, and 57 pre-COVID controls.	- Significant differences in autoreactivity between COVID-19 patients and pre-COVID controls - No patterns of autoreactivity that separated individuals with LC from individuals fully recovered from COVID-19	Absence of LC-specific autoreactivities
Son, K. et al., 2023 [194]	A cohort of 106 convalescent COVID-19 patients with varying acute phase severities at 3-, 6- and 12-months post-recovery, 22 HCs and 34 with other respiratory infections	- Abs to U1-snRNP and anti-SS-B/La were both positively associated with persistent symptoms of fatigue and dyspnea. - Pro-inflammatory cytokines such as TNF-α and CRP predicted ↑ ANAs at 12 months.	Persistently positive ANAs at 12-months post-COVID are associated with persisting symptoms and inflammation in a subset of COVID-19 survivors.
Franke, C. et al., 2023 [195]	A prospective study of 50 patients with reported cognitive problems	- A total of 92% had normal routine CSF parameters. - A total of 52% had antineuronal Abs (n = 9 in serum only, n = 3 in CSF only and n = 14 in both, including those against myelin, Yo, Ma2/Ta, GAD65 and the NMDA receptor along with a variety of undetermined epitopes on brain sections).	An abnormal cognitive status is associated with antineuronal Abs in CSF.
Peluso, M.J. et al., 2022 [196]	A prospective study of 215 participants with convalescent COVID-19 tested over 394 time points, including 121 people with LC	- An amount of 2 out of 215 had IFN-α2-specific autoantibodies across all sample time points.	No detectable anti-IFN-α2 antibodies in LC
Klein, J. et al., 2022 [174]	A total of 220 participants (101 LC, 41 CC, 41 HC and 37 HCW), cross-sectional study	No significant differences in the total number of autoantibody reactivities per participant across groups using REAP, a high throughput method for the measurement of Ab reactivity against >6000 extracellular and secreted human proteins	No specific autoAbs that could differentiate participants with LC from controls
Su, Y. et al., 2022 [190]	A cohort of 309 COVID-19 patients from initial diagnosis to convalescence (2–3 months later), 457 HC Determination of an auto-Ab panel: anti-IFN-α2 and 5 antinuclear auto-Abs (Ro/SS-A, La/SS-B, U1-snRNP, Jo-1 and P1) at clinical diagnosis and convalescence	- Patients with autoAbs at convalescence (44%) already exhibited mature (class-switched) auto-Abs as early as at diagnosis (56%). - Inverse correlations between SARS-CoV-2 IgGs (class-switched) and autoAbs	- IFN-α2 autoAbs uniquely associated with respiratory symptoms of LC - ↑ multiple autoAbs at convalescence are associated with GI symptoms and the sputum production of LC.
Rojas, M. et al., 2022 [197]	Case-control study: 100 patients with LC with a median post-COVID time of 219 (IQR: 143 to 258) days, 30 prepandemic HC	- Latent autoimmunity in 83% of patients - Polyautoimmunity in 62% of patients - Anti-IFN autoAbs in 5–10% of patients - Anti-SARS-CoV-2 IgG correlated with autoAbs	- IgG anti-IFN-λ Abs were associated with the persistence of respiratory symptoms. - Latent autoimmunity correlates with an Ab response against SARS-CoV-2.
***Endothelial or vascular biomarkers***			
Patel, M.A. et al., 2022 [198]	A case-control study of 23 LC patients, one- to six-months post-infection, and 23 ward COVID patients, 23 ICU COVID patients and 23 HCs	- Angiogenesis markers (ANG-1 and P-SEL) had excellent sensitivity and specificity for LC status (AUC = 1.00) among 16 blood biomarkers of vascular transformation. - ANG-1 levels were associated with female sex and a lack of disease interventions at follow-up	- Diagnostic utility and classification of LC status (accuracy: 96%)
Haffke, M. et al., 2022 [199]	A case-control study of 30 LC patients with persistent fatigue and exertion intolerance (14 with 14 post-COVID ME/CFS) and 15 age- and sex matched seronegative HCs	- ↑ ET-1 concentration in both ME/CFS and LC patients compared to HCs and post-convalescent controls - ↓ Ang-2 in both LC patients and post-convalescent controls compared to HCs	- LC patients with fatigue present ↑ ET-1 and ↓ Ang-2 - Ang-2 levels exclusively in LC could be a differentiation biomarker betweenPCS and ME/CFS
Tong, M. et al., 2022 [200]	A cross-sectional study of 345 COVID-19 (39% had LC symptoms) and 119 age and gender-matched HCWs	- No significant differences in vascular biomarkers (serum levels of VCAM-1, ICAM-1, P-selectin) between COVID-19 survivors and controls	
***Biomarkers of coagulation and fibrinolysis***			
Yong, S.J. et al., 2023 [172]	Meta-analysis of 23 studies (14 prospective and 9 retrospective case control) with 18 meta-analyzed biomarkers	- ↑ D-dimer, CRP, LDH and leukocytes in LC patients than those without LC - ↑ lymphocytes and IL-6 in LC patients than those without LC - ↑ IL-8 in LC than healthy controls	- Diagnostic utility in LC; however, the effects are small - ↑ D-dimer, LDH and lymphocytes in patients with organ abnormalities - ↑ D-dimer in patients with duration of symptoms ≥ 6 months
Constantinescu-Bercu, A. et al., 2023 [201]	A cohort of 21 patients with LC with a median time of 23 months of follow-up and controls	- ↑ platelet binding on both collagen and anti-VWF A3 in patients with LC compared with controls, which positively correlated with VWF and VWF(Ag):ADAMTS13 ratio and inversely correlated with ADAMTS13 activity	- LC is a prothrombotic state
Di Gennaro, L. et al., 2022 [202]	A case-control study of 75 children with previously confirmed COVID-19 in a pediatric post-COVID unit ≥ 8 weeks after the initial infection	- ↑ D-dimer levels in LC children compared to children that had fully recovered at the 8–12 weeks (*p* = 0.04) and 12 week follow-ups or more (*p* = 0.05) and in children with three or more symptoms at 12 weeks (*p* = 0.002). - No significant differences in other coagulation factors between LC children and controls	Abnormal D-dimer levels in children with LC and more symptoms
Kruger et al., 2022 [203]	A case-control study of 99 LC patients and 29 HCs.	- ↑ platelet factor 4 (PF4), VWF and α-2 antiplasmin (α-2-AP) in LC - ↓ plasma kallikrein in LC - Significant platelet hyperactivation was noted in LC.	Failed fibrinolytic system in LC
Pretorius, E. et al., 2021 [204]	A cross-sectional study of 11 patients with LC, 13 HCs, 15 with COVID-19 (before treatment) and 10 with T2DM	- Microclots in both acute COVID-19 and LC plasma samples are resistant to fibrinolysis (compared to plasma from controls and T2DM). - ↑ SAA in LC than controls - Platelets from LC patients are hyperactivated. - In the digested clots, ↑ α2 antiplasmin, plasminogen, coagulation factor XIII A chain, vWF, fibrinogen alpha chain, C7 and CRP in LC than controls	Abnormal fibrinolysis and coagulopathy in LC
***Hormonal and metabolic biomarkers***			
di Filippo, L. et al., 2023 [205]	- Total of 50 cases with LC and 50 non-LC subjects matched on a 1:1 basis followed for 6 months. - Follow-up case-control study	- Concentrations of 20.1 vs. 23.2 ng/mL (*p* = 0.03) in cases with LC vs. controls at follow-up - ↓ 25(OH)D in cases with neurocognitive symptoms at follow-up - ↓ 25(OH)D levels at follow-up were the only significant variable in LC	- ↓ 25(OH)D in LC and particularly in those with brain fog - 25(OH)D levels should be evaluated in cases with COVID-19 after hospital discharge.
Mohamed Hussein, A.A.R. et al., 2022 [206]	- A total of 219 post-COVID cases - A cross-sectional, single-center study of all cases attending a post-COVID follow-up clinic	- A proportion of 84% had deficient vitamin D levels (<20 ng/dL). - A proportion of 11.4% had insufficient levels (20–30 ng/dL). - Only 4.9% had normal levels.	- ↑ prevalence of vitamin D deficiency - No association between vitamin D levels and post-COVID symptoms.
Su, Y. et al., 2022 [190]	A cohort of 309 COVID-19 patients from initial diagnosis to convalescence (2–3 months later), 457 HC	- ↓ cortisol and cortisone in patients with LC at convalescence - ↑ proteins associated with the negative regulation of the circadian sleep/wake cycle in patients with neurological symptoms	- Patients with respiratory symptoms at convalescence exhibited ↓ cortisol and cortisone.
Klein, J. et al., 2022 [174]	A total of 220 participants (101 LC, 41 CC, 41 HC and 37 HCW), cross-sectional study	↓ serum cortisol in LC, ↑ in healthy (uninfected) controls, ↓ in convalescent controls and ↓↓ in cases with LC	- Serum cortisol was the most significant individual predictor of LC.
Sunada, N. et al., 2022 [207]	- A total of 186 patients with LC - Retrospective analysis	- ↓ serum GH and ↑ serum FT4 in patients with general fatigue - ↓cortisol in patients with dysosmia/dysgeusia -↑ serum TSH and ↓ ratio of FT4/TSH in initial severe LC cases	
Townsend, L. et al., 2021 [208]	A cohort of 149 patients at a median of 79 days after COVID-19 illness.	- Median vitamin D was 62 nmol/L, with 24% having levels of 30–49 nmol/L (insufficiency) and 9% with levels < 30 nmol/L (deficiency)	- No association between vitamin D and persistent fatigue or decreased exercise tolerance
***Other proteins as biomarkers***			
The PHOSP-COVID Collaborative Group, 2022 [92]	A prospective, longitudinal cohort study: 2320 participants discharged from hospital; 807 (34.7%) participants completed 5-month and 1-year visits.	The plasma proteome data for 296 protein features is as follows: - ↑ of 13 proteins in the severe group of LC.	- ↑ IL-6 and CD70 in cognitive impairment cluster compared with the mild cluster
Captur, G. et al., 2022 [209]	A nested longitudinal proteomic case-control study of 156 healthcare workers	Differentially abundant proteins in HCW with persistent symptoms (>6 w): proteins with lipid, atherosclerosis and cholesterol metabolism pathways, complement and coagulation cascades, autophagy and lysosomal function	Potential predictive value for LC at the time of seroconversion
***Metabolites as biomarkers***			
López-Hernández, Y. et al., 2023 [210]	A longitudinal retrospective analysis of COVID-19 patients (n = 22), LC patients (n = 25) and controls (n = 15)	- Fatigue (59%) and musculoskeletal issues (50%) were most relevant and persistent symptoms. - Sterols, bile acids, isoprenoids and fatty esters were affected in both COVID-19 and post-COVID patients. - ↑ species of phosphatidylcholines and sphingomyelins in LC compared to controls	- Dysregulation in sphingolipid metabolism could be associated with fatigue and muscular pain.
López-Hernández, Y. et al., 2023 [211]	A retrospective longitudinal analysis of 108 participants (HC, COVID-19 and LC patients)	- ↑ Lactic acid, lactate/pyruvate ratio, ornithine/citrulline ratio, sarcosine and arginine in LC - ↑ IL-17 in LC	- Mitochondrial dysfunction, redox state imbalance, impaired energy metabolism and chronic immune dysregulation in LC
Guntur, V.P. et al., 2022 [150]	Case-control study of 29 patients with LC, 16 CC and 30 HC	- ↑ free- and carnitine-conjugated mono-, poly- and highly unsaturated fatty acids and ↓ levels of mono-, di- and tricarboxylates, polyamines (spermine) and taurine in LC - Milder disturbances in fatty acid metabolism and ↑ spermine and taurine in recovered patients - Tryptophan depletion not normalized in LC	- Altered fatty acid metabolism and dysfunctional mitochondria-dependent lipid catabolism associated with mitochondrial dysfunction during exercise
Cysique, L.A. et al., 2022 [212]	A prospective study of 128 SARS-CoV-2-positive patients	- ↑ quinolinic acid, 3-hydroxyanthranilic acid and kynurenine were significantly associated with cognitive decline.	The kynurenine pathway metabolites are potential therapeutic targets for COVID-19-related cognitive impairment.
***Microbiota alterations in long COVID***			
Zhang, D. et al., 2023 [213]	A prospective follow-up study of 187 recovered subjects; 84 reported LC symptoms at one-year after discharge; 32 HCs, 16S rRNA sequencing of stool samples	- Gut microbiota dysbiosis in symptomatic recovered patients - ↓ bacterial diversities in LC - ↓ relative abundance of SCFA-producing salutary symbionts, such as *Eubacterium hallii*, *Subdoligranulum*, *Ruminococcus*, *Dorea*, *Coprococcus* and *Eubacterium_ventriosum* groups in LC	- Gut microbiota dysbiosis in recovered patients at one-year after discharge - Gut microbiota dysbiosis in LC
Liu, Q. et al., 2022 [214]	A prospective study of 106 patients with a spectrum of COVID-19 severity from admission to 6 months and 68 non-COVID-19 controls	- ↑ *Ruminococcus gnavus, Bacteroides vulgatus* in LC - ↓ *Faecalibacterium prausnitzii* in LC - ↓ Butyrate-producing bacteria in LC	- Gut microbiota composition at admission was predictive of LC occurrence.
***Cerebrospinal fluid biomarkers***			
Etter, M.M. et al., 2022 [142]	A cohort study of 40 neuro-COVID patients; 25 HCs and 25 non-MS inflammatory neurological disease controls	- CSF levels: ↑ pro-inflammatory proteins (TNFRSF9, IFN-γ) and ↓ anti-inflammatory mediators (TRANCE(RANKL), TRAIL) are predictive for LC. - plasma CLM-6, MCP-3 and ST1A1 may predict LC.	- Prediction of LC
Apple, A.C. et al., 2022 [215]	A case-control study of 22 participants with cognitive LC and 10 cognitive controls within the prospective study LIINC	- CSF abnormality in 77% of LC patients versus 0% of cognitive controls - Normal values for CSF white blood cells, glucose, calculated CSF/serum albumin ratio, IgG index, CSF IgG level and serum IgG level in all participants - Abnormal oligoclonal banding in 69% of LC patients versus 0% of cognitive controls	- ↑% of CSF abnormalities in patients with LC and cognitive impairment after mild COVID-19
Guasp, M. et al., 2022 [216]	A prospective study of 60 hospitalized neuro-COVID patients, 25 of them with encephalopathy and 14 with encephalitis. A total of 46 serum samples from HCs and 24 CSF samples from subjects with mild subjective cognitive complaints. Follow-up: 18 months	- ↑ IL-18, IL-6 and IL-8 in both serum and CSF in neuro-COVID patients compared to HCs - ↑ 14-3-3, NfL, IL-18, IL-1RA and IL-8 are associated with acute COVID-19 severity - ↑ CSF 14-3-3 and NfL correlate with the degree of neurologic disability in the daily activities at 18 months	- ↑ CSF levels of neuronal damage biomarkers during the acute phase of COVID-19 are prognostic biomarkers of worse long-term clinical outcomes of patients.

Abbreviations: ↑, increased; ↓, decreased; Ab, antibody; ANAs, antinuclear antibodies; Ang-2, Angiopoietin-2; CC, convalescent control; CD, dendritic cell; CIs, confidence intervals; CNS, central nervous system; CSF, cerebrospinal fluid; EBV, Epstein–Barr Virus; EBNA, EBV-encoded nuclear antigen; ET-1, endothelin-1; HC, healthy control; FT4, free thyroxine; GH, growth hormone; GI, gastrointestinal; HHV, Human Herpesvirus; HOMA-IR, Homeostasis Model Assessment-Insulin Resistance; HW, healthcare worker; IBD, inflammatory bowel disease; ICAM-1, Intercellular Adhesion Molecule-1; IFN, interferon; IL, interleukin; IQR, interquartile range; LC, long COVID; LDH, lactate dehydrogenase; MIS-C, Multisystem Inflammatory Syndrome in children; NfL, neurofilament light chain protein; NK, natural killer cell; NT-Pro-BNP, N-terminal pro b-type natriuretic peptide; PASC, post-acute sequelae SARS-CoV-2 infection; REAP, Rapid Extracellular Antigen Profiling; SCFAs, short-chain fatty acids; T2DM, diabetes mellitus type 2; VCA, viral capsid antigen; VCAM, Vascular Cell Adhesion Molecule 1;VEGF, Vascular Endothelial Growth Factor; VWF, Von Willebrand factor.

## Data Availability

Not applicable.

## References

[1] WHO Coronavirus (COVID-19) Dashboard. https://covid19.who.int/.

[2] Lee W.E., Woo Park S., Weinberger D.M., Olson D., Simonsen L., Grenfell B.T., Viboud C. (2023). Direct and indirect mortality impacts of the COVID-19 pandemic in the United States, 1 March 2020 to 1 January 2022. Elife.

[3] Nicola M., Alsafi Z., Sohrabi C., Kerwan A., Al-Jabir A., Iosifidis C., Agha M., Agha R. (2020). The socio-economic implications of the coronavirus pandemic (COVID-19): A review. Int. J. Surg..

[4] Tsilingiris D., Vallianou N.G., Karampela I., Liu J., Dalamaga M. (2022). Potential implications of lipid nanoparticles in the pathogenesis of myocarditis associated with the use of mRNA vaccines against SARS-CoV-2. Metab. Open.

[5] Marzianο V., Guzzetta G., Menegale F., Sacco C., Petrone D., Urdiales A., del Manso M., Bella A., Fabiani M., Vescio M. (2022). The decline of COVID-19 severity and lethality over two years of pandemic. Res. Sq..

[6] Sigal A. (2022). Milder disease with Omicron: Is it the virus or the pre-existing immunity?. Nat. Rev. Immunol..

[7] Mahase E. (2023). COVID-19: What do we know about XBB.1.5 and should we be worried?. BMJ.

[8] WHO XBB.1.5 Updated Risk Assessment, 24 February 2023. https://www.who.int/docs/default-source/coronaviruse/22022024xbb.1.5ra.pdf.

[9] Siddiqui S., Alhamdi H.W.S., Alghamdi H.A. (2022). Recent Chronology of COVID-19 Pandemic. Front. Public Health.

[10] Líška D., Liptaková E., Babičová A., Batalik L., Baňárová P.S., Dobrodenková S. (2022). What is the quality of life in patients with long COVID compared to a healthy control group?. Front. Public Health.

[11] Davis H.E., McCorkell L., Vogel J.M., Topol E.J. (2023). Long COVID: Major findings, mechanisms and recommendations. Nat. Rev. Microbiol..

[12] WHO: Post COVID-19 Condition (Long COVID). https://www.who.int/europe/news-room/fact-sheets/item/post-covid-19-condition#:~:text=It%20is%20defined%20as%20the,months%20with%20no%20other%20explanation.

[13] NICE: COVID-19 Rapid Guideline: Managing the Longterm Effects of COVID-19. https://www.nice.org.uk/guidance/ng188/resources/covid19-rapid-guideline-managing-the-longterm-effects-of-covid19-pdf-51035515742#:~:text=In%20addition%20to%20the%20clinical,)%20and%20post%E2%80%91COVID%E2%80%9119.

[14] CDC: Long COVID or Post-COVID Conditions. https://www.cdc.gov/coronavirus/2019-ncov/long-term-effects/index.html#:~:text=Long%20COVID%20is%20broadly%20defined,with%20CDC%20and%20other%20partners.

[15] Robert Koch Institute: RKI Information Portal on Long COVID. https://www.rki.de/EN/Content/infections/epidemiology/outbreaks/COVID-19/Long-COVID/content-total.html.

[16] Government of Canada: Post COVID-19 Condition (Long COVID). https://www.canada.ca/en/public-health/services/diseases/2019-novel-coronavirus-infection/symptoms/post-covid-19-condition.html.

[17] Jennings G., Monaghan A., Xue F., Mockler D., Romero-Ortuño R. (2021). A Systematic Review of Persistent Symptoms and Residual Abnormal Functioning following Acute COVID-19: Ongoing Symptomatic Phase vs. Post-COVID-19 Syndrome. J. Clin. Med..

[18] WHO: Expanding Our Understanding of Post COVID-19 Condition Report of a WHO Webinar, 9 February 2021. https://www.who.int/publications/i/item/9789240025035.

[19] Roe K. (2021). The Symptoms and Clinical Manifestations Observed in COVID-19 Patients/Long COVID-19 Symptoms that Parallel Toxoplasma gondii Infections. J. Neuroimmune Pharmacol..

[20] Lopez-Leon S., Wegman-Ostrosky T., Perelman C., Sepulveda R., Rebolledo P.A., Cuapio A., Villapol S. (2021). More than 50 long-term effects of COVID-19: A systematic review and meta-analysis. Sci. Rep..

[21] Castanares-Zapatero D., Chalon P., Kohn L., Dauvrin M., Detollenaere J., Maertens de Noordhout C., Primus-de Jong C., Cleemput I., Van den Heede K. (2022). Pathophysiology and mechanism of long COVID: A comprehensive review. Ann. Med..

[22] Dalamaga M., Karmaniolas K., Matekovits A., Migdalis I., Papadavid E. (2008). Cutaneous manifestations in relation to immunologic parameters in a cohort of primary myelodysplastic syndrome patients. J. Eur. Acad. Dermatol. Venereol..

[23] Mentis A.A., Dalamaga M., Lu C., Polissiou M.G. (2021). Saffron for “toning down” COVID-19-related cytokine storm: Hype or hope? A mini-review of current evidence. Metab. Open.

[24] Varghese J., Sandmann S., Ochs K., Schrempf I.M., Frömmel C., Dugas M., Schmidt H.H., Vollenberg R., Tepasse P.R. (2021). Persistent symptoms and lab abnormalities in patients who recovered from COVID-19. Sci. Rep..

[25] Espín E., Yang C., Shannon C.P., Assadian S., He D., Tebbutt S.J. (2023). Cellular and molecular biomarkers of long COVID: A scoping review. EBioMedicine.

[26] Lai Y.J., Liu S.H., Manachevakul S., Lee T.A., Kuo C.T., Bello D. (2023). Biomarkers in long COVID-19: A systematic review. Front. Med..

[27] Nasserie T., Hittle M., Goodman S.N. (2021). Assessment of the Frequency and Variety of Persistent Symptoms among Patients with COVID-19: A Systematic Review. JAMA Netw. Open.

[28] Mizrahi B., Sudry T., Flaks-Manov N., Yehezkelli Y., Kalkstein N., Akiva P., Ekka-Zohar A., Ben David S.S., Lerner U., Bivas-Benita M. (2023). Long COVID outcomes at one year after mild SARS-CoV-2 infection: Nationwide cohort study. BMJ.

[29] UK Office for National Statistics Prevalence of Ongoing Symptoms Following Coronavirus (COVID-19) Infection in the UK. https://www.ons.gov.uk/peoplepopulationandcommunity/healthandsocialcare/conditionsanddiseases/datasets/alldatarelatingtoprevalenceofongoingsymptomsfollowingcoronaviruscovid19infectionintheuk.

[30] Ahmad F.B., Anderson R.N., Cisewski J.A., Sutton P.D. (2022). Identification of Deaths with Post-Acute Sequelae of COVID-19 from death Certificate Literal Text: United States, 1 January 2020–30 June 2022.

[31] Du M., Ma Y., Deng J., Liu M., Liu J. (2022). Comparison of Long COVID-19 Caused by Different SARS-CoV-2 Strains: A Systematic Review and Meta-Analysis. Int. J. Environ. Res. Public Health.

[32] Antonelli M., Pujol J.C., Spector T.D., Ourselin S., Steves C.J. (2022). Risk of long COVID associated with delta versus omicron variants of SARS-CoV-2. Lancet.

[33] Nehme M., Vetter P., Chappuis F., Kaiser L., Guessous I. (2023). Prevalence of Post-Coronavirus Disease Condition 12 Weeks after Omicron Infection Compared with Negative Controls and Association with Vaccination Status. Clin. Infect. Dis..

[34] Ballouz T., Menges D., Kaufmann M., Amati R., Frei A., von Wyl V., Fehr J.S., Albanese E., Puhan M.A. (2023). Post COVID-19 condition after Wildtype, Delta, and Omicron SARS-CoV-2 infection and prior vaccination: Pooled analysis of two population-based cohorts. PLoS ONE.

[35] Levy N., Koppel J.H., Kaplan O., Yechiam H., Shahar-Nissan K., Cohen N.K., Shavit I. (2022). Severity and Incidence of Multisystem Inflammatory Syndrome in Children during 3 SARS-CoV-2 Pandemic Waves in Israel. JAMA.

[36] Amanatidou E., Gkiouliava A., Pella E., Serafidi M., Tsilingiris D., Vallianou N.G., Karampela I., Dalamaga M. (2022). Breakthrough infections after COVID-19 vaccination: Insights, perspectives and challenges. Metab. Open.

[37] Dalamaga M., Nasiri-Ansari N., Spyrou N. (2023). Perspectives and Challenges of COVID-19 with Obesity-Related Cancers. Cancers.

[38] Tsilingiris D., Nasiri-Ansari N., Spyrou N., Magkos F., Dalamaga M. (2022). Management of Hematologic Malignancies in the Era of COVID-19 Pandemic: Pathogenetic Mechanisms, Impact of Obesity, Perspectives, and Challenges. Cancers.

[39] Syriga M., Karampela I., Dalamaga M., Karampelas M. (2021). The effect of COVID-19 pandemic on the attendance and clinical outcomes of patients with ophthalmic disease: A mini-review. Metab. Open.

[40] Kikkenborg Berg S., Palm P., Nygaard U., Bundgaard H., Petersen M.N.S., Rosenkilde S., Thorsted A.B., Ersbøll A.K., Thygesen L.C., Nielsen S.D. (2022). Long COVID symptoms in SARS-CoV-2-positive children aged 0-14 years and matched controls in Denmark (LongCOVIDKidsDK): A national, cross-sectional study. Lancet. Child Adolesc. Health.

[41] Sørensen A.I.V., Spiliopoulos L., Bager P., Nielsen N.M., Hansen J.V., Koch A., Meder I.K., Ethelberg S., Hviid A. (2022). A nationwide questionnaire study of post-acute symptoms and health problems after SARS-CoV-2 infection in Denmark. Nat. Commun..

[42] Roessler M., Tesch F., Batram M., Jacob J., Loser F., Weidinger O., Wende D., Vivirito A., Toepfner N., Ehm F. (2022). Post-COVID-19-associated morbidity in children, adolescents, and adults: A matched cohort study including more than 157,000 individuals with COVID-19 in Germany. PLoS Med..

[43] Edlow A.G., Castro V.M., Shook L.L., Kaimal A.J., Perlis R.H. (2022). Neurodevelopmental Outcomes at 1 Year in Infants of Mothers Who Tested Positive for SARS-CoV-2 during Pregnancy. JAMA Netw. Open.

[44] Vella L.A., Rowley A.H. (2021). Current Insights Into the Pathophysiology of Multisystem Inflammatory Syndrome in Children. Curr. Pediatr. Rep..

[45] Melgar M., Lee E.H., Miller A.D., Lim S., Brown C.M., Yousaf A.R., Zambrano L.D., Belay E.D., Godfred-Cato S., Abrams J.Y. (2022). Council of State and Territorial Epidemiologists/CDC Surveillance Case Definition for Multisystem Inflammatory Syndrome in Children Associated with SARS-CoV-2 Infection—United States. MMWR. Recomm. Rep..

[46] Tsampasian V., Elghazaly H., Chattopadhyay R., Debski M., Naing T.K.P., Garg P., Clark A., Ntatsaki E., Vassiliou V.S. (2023). Risk Factors Associated with Post-COVID-19 Condition: A Systematic Review and Meta-analysis. JAMA Intern. Med..

[47] Asadi-Pooya A.A., Akbari A., Emami A., Lotfi M., Rostamihosseinkhani M., Nemati H., Barzegar Z., Kabiri M., Zeraatpisheh Z., Farjoud-Kouhanjani M. (2021). Risk Factors Associated with Long COVID Syndrome: A Retrospective Study. Iran. J. Med. Sci..

[48] Subramanian A., Nirantharakumar K., Hughes S., Myles P., Williams T., Gokhale K.M., Taverner T., Chandan J.S., Brown K., Simms-Williams N. (2022). Symptoms and risk factors for long COVID in non-hospitalized adults. Nat. Med..

[49] Rao S., Lee G.M., Razzaghi H., Lorman V., Mejias A., Pajor N.M., Thacker D., Webb R., Dickinson K., Bailey L.C. (2022). Clinical Features and Burden of Postacute Sequelae of SARS-CoV-2 Infection in Children and Adolescents. JAMA Pediatr..

[50] Morello R., Mariani F., Mastrantoni L., De Rose C., Zampino G., Munblit D., Sigfrid L., Valentini P., Buonsenso D. (2023). Risk factors for post-COVID-19 condition (Long COVID) in children: A prospective cohort study. EClinicalMedicine.

[51] Yu Z., Ekström S., Bellander T., Ljungman P., Pershagen G., Eneroth K., Kull I., Bergström A., Georgelis A., Stafoggia M. (2023). Ambient air pollution exposure linked to long COVID among young adults: A nested survey in a population-based cohort in Sweden. Lancet Reg. Health. Eur..

[52] Merzon E., Weiss M., Krone B., Cohen S., Ilani G., Vinker S., Cohen-Golan A., Green I., Israel A., Schneider T. (2022). Clinical and Socio-Demographic Variables Associated with the Diagnosis of Long COVID Syndrome in Youth: A Population-Based Study. Int. J. Environ. Res. Public Health.

[53] Ayoubkhani D., Bosworth M.L., King S., Pouwels K.B., Glickman M., Nafilyan V., Zaccardi F., Khunti K., Alwan N.A., Walker A.S. (2022). Risk of Long COVID in People Infected with Severe Acute Respiratory Syndrome Coronavirus 2 After 2 Doses of a Coronavirus Disease 2019 Vaccine: Community-Based, Matched Cohort Study. Open Forum Infect. Dis..

[54] Al-Aly Z., Bowe B., Xie Y. (2022). Long COVID after breakthrough SARS-CoV-2 infection. Nat. Med..

[55] Antar A.A.R., Yu T., Demko Z.O., Hu C., Tornheim J.A., Blair P.W., Thomas D.L., Manabe Y.C. (2023). Long COVID brain fog and muscle pain are associated with longer time to clearance of SARS-CoV-2 RNA from the upper respiratory tract during acute infection. medRxiv.

[56] Tsuchida T., Hirose M., Inoue Y., Kunishima H., Otsubo T., Matsuda T. (2022). Relationship between changes in symptoms and antibody titers after a single vaccination in patients with Long COVID. J. Med. Virol..

[57] Nguyen N.N., Nguyen Y.N., Hoang V.T., Million M., Gautret P. (2023). SARS-CoV-2 Reinfection and Severity of the Disease: A Systematic Review and Meta-Analysis. Viruses.

[58] Bowe B., Xie Y., Al-Aly Z. (2022). Acute and postacute sequelae associated with SARS-CoV-2 reinfection. Nat. Med..

[59] Chen C., Haupert S.R., Zimmermann L., Shi X., Fritsche L.G., Mukherjee B. (2022). Global Prevalence of Post-Coronavirus Disease 2019 (COVID-19) Condition or Long COVID: A Meta-Analysis and Systematic Review. J. Infect. Dis..

[60] Izquierdo-Pujol J., Moron-Lopez S., Dalmau J., Gonzalez-Aumatell A., Carreras-Abad C., Mendez M., Rodrigo C., Martinez-Picado J. (2022). Post COVID-19 Condition in Children and Adolescents: An Emerging Problem. Front. Pediatr..

[61] Gonzalez-Aumatell A., Bovo M.V., Carreras-Abad C., Cuso-Perez S., Domènech Marsal È., Coll-Fernández R., Goicoechea Calvo A., Giralt-López M., Enseñat Cantallops A., Moron-Lopez S. (2022). Social, Academic, and Health Status Impact of Long COVID on Children and Young People: An Observational, Descriptive, and Longitudinal Cohort Study. Children.

[62] Fonseca A., Lima R., Ladeira I., Guimarães M. (2021). Evaluation of pulmonary function in post-COVID-19 patients—When and how should we do it?. J. Bras. De Pneumol..

[63] Fortini A., Rosso A., Cecchini P., Torrigiani A., Lo Forte A., Carrai P., Alessi C., Fabbrizzi F., Lovicu E., Sbaragli S. (2022). One-year evolution of DLCO changes and respiratory symptoms in patients with post COVID-19 respiratory syndrome. Infection.

[64] Kanne J.P., Little B.P., Schulte J.J., Haramati A., Haramati L.B. (2023). Long-term Lung Abnormalities Associated with COVID-19 Pneumonia. Radiology.

[65] Michelen M., Manoharan L., Elkheir N., Cheng V., Dagens A., Hastie C., O’Hara M., Suett J., Dahmash D., Bugaeva P. (2021). Characterising long COVID: A living systematic review. BMJ Glob. Health.

[66] Canas L.S., Molteni E., Deng J., Sudre C.H., Murray B., Kerfoot E., Antonelli M., Chen L., Rjoob K., Pujol J.C. (2022). Profiling post-COVID syndrome across different variants of SARS-CoV-2. medRxiv.

[67] Dennis A., Cuthbertson D.J., Wootton D., Crooks M., Gabbay M., Eichert N., Mouchti S., Pansini M., Roca-Fernandez A., Thomaides-Brears H. (2023). Multi-organ impairment and long COVID: A 1-year prospective, longitudinal cohort study. J. R. Soc. Med..

[68] Fedorowski A., Sutton R. (2023). Autonomic dysfunction and postural orthostatic tachycardia syndrome in post-acute COVID-19 syndrome. Nat. Rev. Cardiol..

[69] Espinosa-Gonzalez A.B., Master H., Gall N., Halpin S., Rogers N., Greenhalgh T. (2023). Orthostatic tachycardia after COVID-19. BMJ.

[70] Agashe S., Petak S. (2018). Cardiac Autonomic Neuropathy in Diabetes Mellitus. Methodist DeBakey Cardiovasc. J..

[71] Guo B., Zhao C., He M.Z., Senter C., Zhou Z., Peng J., Li S., Fitzpatrick A.L., Lindström S., Stebbins R.C. (2023). Long-term cardiac symptoms following COVID-19: A systematic review and meta-analysis. medRxiv.

[72] Xie Y., Xu E., Bowe B., Al-Aly Z. (2022). Long-term cardiovascular outcomes of COVID-19. Nat. Med..

[73] Harding J.L., Oviedo S.A., Ali M.K., Ofotokun I., Gander J.C., Patel S.A., Magliano D.J., Patzer R.E. (2023). The bidirectional association between diabetes and long-COVID-19—A systematic review. Diabetes Res. Clin. Pract..

[74] Nassar M., Daoud A., Nso N., Medina L., Ghernautan V., Bhangoo H., Nyein A., Mohamed M., Alqassieh A., Soliman K. (2021). Diabetes Mellitus and COVID-19: Review Article. Diabetes Metab. Syndr..

[75] Karampela I., Vallianou N., Magkos F., Apovian C.M., Dalamaga M. (2022). Obesity, Hypovitaminosis D, and COVID-19: The Bermuda Triangle in Public Health. Curr. Obes. Rep..

[76] Dalamaga M., Christodoulatos G.S., Karampela I., Vallianou N., Apovian C.M. (2021). Understanding the Co-Epidemic of Obesity and COVID-19: Current Evidence, Comparison with Previous Epidemics, Mechanisms, and Preventive and Therapeutic Perspectives. Curr. Obes. Rep..

[77] Vallianou N.G., Evangelopoulos A., Kounatidis D., Stratigou T., Christodoulatos G.S., Karampela I., Dalamaga M. (2021). Diabetes Mellitus and SARS-CoV-2 Infection: Pathophysiologic Mechanisms and Implications in Management. Curr. Diabetes Rev..

[78] Al-Aly Z. (2023). Diabetes after SARS-CoV-2 infection. Lancet. Diabetes Endocrinol..

[79] Kendall E.K., Olaker V.R., Kaelber D.C., Xu R., Davis P.B. (2022). Association of SARS-CoV-2 Infection with New-Onset Type 1 Diabetes among Pediatric Patients from 2020 to 2021. JAMA Netw. Open.

[80] Tsilingiris D., Dalamaga M., Liu J. (2022). SARS-CoV-2 adipose tissue infection and hyperglycemia: A further step towards the understanding of severe COVID-19. Metab. Open.

[81] Tansey C.M., Louie M., Loeb M., Gold W.L., Muller M.P., de Jager J., Cameron J.I., Tomlinson G., Mazzulli T., Walmsley S.L. (2007). One-year outcomes and health care utilization in survivors of severe acute respiratory syndrome. Arch. Intern. Med..

[82] Lam M.H., Wing Y.K., Yu M.W., Leung C.M., Ma R.C., Kong A.P., So W.Y., Fong S.Y., Lam S.P. (2009). Mental morbidities and chronic fatigue in severe acute respiratory syndrome survivors: Long-term follow-up. Arch. Intern. Med..

[83] Lee S.H., Shin H.S., Park H.Y., Kim J.L., Lee J.J., Lee H., Won S.D., Han W. (2019). Depression as a Mediator of Chronic Fatigue and Post-Traumatic Stress Symptoms in Middle East Respiratory Syndrome Survivors. Psychiatry Investig..

[84] Moldofsky H., Patcai J. (2011). Chronic widespread musculoskeletal pain, fatigue, depression and disordered sleep in chronic post-SARS syndrome; a case-controlled study. BMC Neurol..

[85] Fukuda K., Straus S.E., Hickie I., Sharpe M.C., Dobbins J.G., Komaroff A., International Chronic Fatigue Syndrome Study Group (1994). The chronic fatigue syndrome: A comprehensive approach to its definition and study. Ann. Intern. Med..

[86] CDC Myalgic Encephalomyelitis/Chronic Fatigue Syndrome: Symptoms. https://www.cdc.gov/me-cfs/symptoms-diagnosis/symptoms.html.

[87] Magnus P., Gunnes N., Tveito K., Bakken I.J., Ghaderi S., Stoltenberg C., Hornig M., Lipkin W.I., Trogstad L., Håberg S.E. (2015). Chronic fatigue syndrome/myalgic encephalomyelitis (CFS/ME) is associated with pandemic influenza infection, but not with an adjuvanted pandemic influenza vaccine. Vaccine.

[88] Wilson H.W., Amo-Addae M., Kenu E., Ilesanmi O.S., Ameme D.K., Sackey S.O. (2018). Post-Ebola Syndrome among Ebola Virus Disease Survivors in Montserrado County, Liberia 2016. BioMed Res. Int..

[89] Garcia M.N., Hause A.M., Walker C.M., Orange J.S., Hasbun R., Murray K.O. (2014). Evaluation of prolonged fatigue post-West Nile virus infection and association of fatigue with elevated antiviral and proinflammatory cytokines. Viral Immunol..

[90] Shikova E., Reshkova V., Kumanova A., Raleva S., Alexandrova D., Capo N., Murovska M. (2020). Cytomegalovirus, Epstein-Barr virus, and human herpesvirus-6 infections in patients with myalgic encephalomyelitis/chronic fatigue syndrome. J. Med. Virol..

[91] Lim E.J., Ahn Y.C., Jang E.S., Lee S.W., Lee S.H., Son C.G. (2020). Systematic review and meta-analysis of the prevalence of chronic fatigue syndrome/myalgic encephalomyelitis (CFS/ME). J. Transl. Med..

[92] (2022). Clinical characteristics with inflammation profiling of long COVID and association with 1-year recovery following hospitalisation in the UK: A prospective observational study. Lancet. Respir. Med..

[93] Kritas S.K., Ronconi G., Caraffa A., Gallenga C.E., Ross R., Conti P. (2020). Mast cells contribute to coronavirus-induced inflammation: New anti-inflammatory strategy. J. Biol. Regul. Homeost. Agents.

[94] Turner S., Khan M.A., Putrino D., Woodcock A., Kell D.B., Pretorius E. (2023). Long COVID: Pathophysiological factors and abnormalities of coagulation. Trends Endocrinol. Metab..

[95] Hu F., Chen F., Ou Z., Fan Q., Tan X., Wang Y., Pan Y., Ke B., Li L., Guan Y. (2020). A compromised specific humoral immune response against the SARS-CoV-2 receptor-binding domain is related to viral persistence and periodic shedding in the gastrointestinal tract. Cell. Mol. Immunol..

[96] Phetsouphanh C., Darley D.R., Wilson D.B., Howe A., Munier C.M.L., Patel S.K., Juno J.A., Burrell L.M., Kent S.J., Dore G.J. (2022). Immunological dysfunction persists for 8 months following initial mild-to-moderate SARS-CoV-2 infection. Nat. Immunol..

[97] Pinho J.R.R., Oliveira K.G., Sitnik R., Maluf M.M., Rodrigues P.H.S., Santana R.A.F., Welter E.R., Irony O. (2021). Long term persistence of coronavirus SARS-CoV-2 infection. Einstein.

[98] Stein S.R., Ramelli S.C., Grazioli A., Chung J.Y., Singh M., Yinda C.K., Winkler C.W., Sun J., Dickey J.M., Ylaya K. (2022). SARS-CoV-2 infection and persistence in the human body and brain at autopsy. Nature.

[99] Karn V., Ahmed S., Tsai L.W., Dubey R., Ojha S., Singh H.N., Kumar M., Gupta P.K., Sadhu S., Jha N.K. (2021). Extracellular Vesicle-Based Therapy for COVID-19: Promises, Challenges and Future Prospects. Biomedicines.

[100] Vojdani A., Vojdani E., Saidara E., Maes M. (2023). Persistent SARS-CoV-2 Infection, EBV, HHV-6 and Other Factors May Contribute to Inflammation and Autoimmunity in Long COVID. Viruses.

[101] García-Abellán J., Padilla S., Fernández-González M., García J.A., Agulló V., Andreo M., Ruiz S., Galiana A., Gutiérrez F., Masiá M. (2021). Antibody Response to SARS-CoV-2 is Associated with Long-term Clinical Outcome in Patients with COVID-19: A Longitudinal Study. J. Clin. Immunol..

[102] Spatola M., Nziza N., Jung W., Deng Y., Yuan D., Dinoto A., Bozzetti S., Chiodega V., Ferrari S., Lauffenburger D.A. (2023). Neurologic sequelae of COVID-19 are determined by immunologic imprinting from previous coronaviruses. Brain.

[103] Proal A.D., VanElzakker M.B. (2021). Long COVID or Post-acute Sequelae of COVID-19 (PASC): An Overview of Biological Factors That May Contribute to Persistent Symptoms. Front. Microbiol..

[104] Sausen D.G., Basith A., Muqeemuddin S. (2023). EBV and Lymphomagenesis. Cancers.

[105] Lupo J., Truffot A., Andreani J., Habib M., Epaulard O., Morand P., Germi R. (2023). Virological Markers in Epstein-Barr Virus-Associated Diseases. Viruses.

[106] Lanz T.V., Brewer R.C., Ho P.P., Moon J.S., Jude K.M., Fernandez D., Fernandes R.A., Gomez A.M., Nadj G.S., Bartley C.M. (2022). Clonally expanded B cells in multiple sclerosis bind EBV EBNA1 and GlialCAM. Nature.

[107] Du Toit A. (2022). EBV linked to multiple sclerosis. Nat. Rev. Microbiol..

[108] Vallianou N.G., Tsilingiris D., Karampela I., Liu J., Dalamaga M. (2022). Herpes zoster following COVID-19 vaccination in an immunocompetent and vaccinated for herpes zoster adult: A two-vaccine related event?. Metab. Open.

[109] Argyrakopoulou G., Dalamaga M., Spyrou N., Kokkinos A. (2021). Gender Differences in Obesity-Related Cancers. Curr. Obes. Rep..

[110] O’Leary K. (2022). Mounting evidence for EBV links to multiple sclerosis. Nat. Med..

[111] Nekoua M.P., Alidjinou E.K., Hober D. (2022). Persistent coxsackievirus B infection and pathogenesis of type 1 diabetes mellitus. Nat. Rev. Endocrinol..

[112] Wang L., Cao Z.M., Zhang L.L., Dai X.C., Liu Z.J., Zeng Y.X., Li X.Y., Wu Q.J., Lv W.L. (2022). Helicobacter Pylori and Autoimmune Diseases: Involving Multiple Systems. Front. Immunol..

[113] Zangiabadian M., Mirsaeidi M., Pooyafar M.H., Goudarzi M., Nasiri M.J. (2021). Associations of Yersinia Enterocolitica Infection with Autoimmune Thyroid Diseases: A Systematic Review and Meta-Analysis. Endocr. Metab. Immune Disord. Drug Targets.

[114] Iwasaki A., Putrino D. (2023). Why we need a deeper understanding of the pathophysiology of long COVID. Lancet. Infect. Dis..

[115] Lake C.M., Breen J.J. (2023). Sequence similarity between SARS-CoV-2 nucleocapsid and multiple sclerosis-associated proteins provides insight into viral neuropathogenesis following infection. Sci. Rep..

[116] Tsilingiris D., Vallianou N.G., Karampela I., Dalamaga M. (2021). Vaccine induced thrombotic thrombocytopenia: The shady chapter of a success story. Metab. Open.

[117] Brogna C., Brogna B., Bisaccia D.R., Lauritano F., Marino G., Montano L., Cristoni S., Prisco M., Piscopo M. (2022). Could SARS-CoV-2 Have Bacteriophage Behavior or Induce the Activity of Other Bacteriophages?. Vaccines.

[118] Zuo T., Zhang F., Lui G.C.Y., Yeoh Y.K., Li A.Y.L., Zhan H., Wan Y., Chung A.C.K., Cheung C.P., Chen N. (2020). Alterations in Gut Microbiota of Patients with COVID-19 during Time of Hospitalization. Gastroenterology.

[119] Vallianou N., Kounatidis D., Christodoulatos G.S., Panagopoulos F., Karampela I., Dalamaga M. (2021). Mycobiome and Cancer: What Is the Evidence?. Cancers.

[120] Spyrou N., Vallianou N., Kadillari J., Dalamaga M. (2021). The interplay of obesity, gut microbiome and diet in the immune check point inhibitors therapy era. Semin. Cancer Biol..

[121] Vallianou N., Dalamaga M., Stratigou T., Karampela I., Tsigalou C. (2021). Do Antibiotics Cause Obesity Through Long-term Alterations in the Gut Microbiome? A Review of Current Evidence. Curr. Obes. Rep..

[122] Tsigalou C., Vallianou N., Dalamaga M. (2020). Autoantibody Production in Obesity: Is There Evidence for a Link Between Obesity and Autoimmunity?. Curr. Obes. Rep..

[123] Koliaki C., Liatis S., Dalamaga M., Kokkinos A. (2020). The Implication of Gut Hormones in the Regulation of Energy Homeostasis and Their Role in the Pathophysiology of Obesity. Curr. Obes. Rep..

[124] Ahamed J., Laurence J. (2022). Long COVID endotheliopathy: Hypothesized mechanisms and potential therapeutic approaches. J. Clin. Investig..

[125] Paniz-Mondolfi A., Bryce C., Grimes Z., Gordon R.E., Reidy J., Lednicky J., Sordillo E.M., Fowkes M. (2020). Central nervous system involvement by severe acute respiratory syndrome coronavirus-2 (SARS-CoV-2). J. Med. Virol..

[126] Fox S.E., Li G., Akmatbekov A., Harbert J.L., Lameira F.S., Brown J.Q., Vander Heide R.S. (2020). Unexpected Features of Cardiac Pathology in COVID-19 Infection. Circulation.

[127] Wang D., Hu B., Hu C., Zhu F., Liu X., Zhang J., Wang B., Xiang H., Cheng Z., Xiong Y. (2020). Clinical Characteristics of 138 Hospitalized Patients with 2019 Novel Coronavirus-Infected Pneumonia in Wuhan, China. Jama.

[128] Kassi E., Dalamaga M., Hroussalas G., Kazanis K., Merantzi G., Zachari A., Giamarellos-Bourboulis E.J., Dionyssiou-Asteriou A. (2010). Adipocyte factors, high-sensitive C-reactive protein levels and lipoxidative stress products in overweight postmenopausal women with normal and impaired OGTT. Maturitas.

[129] Hroussalas G., Kassi E., Dalamaga M., Delimaris I., Zachari A., Dionyssiou-Asteriou A. (2008). Leptin, soluble leptin receptor, adiponectin and resistin in relation to OGTT in overweight/obese postmenopausal women. Maturitas.

[130] Tsilingiris D., Tzeravini E., Koliaki C., Dalamaga M., Kokkinos A. (2021). The Role of Mitochondrial Adaptation and Metabolic Flexibility in the Pathophysiology of Obesity and Insulin Resistance: An Updated Overview. Curr. Obes. Rep..

[131] Kassi E., Dalamaga M., Faviou E., Hroussalas G., Kazanis K., Nounopoulos C., Dionyssiou-Asteriou A. (2009). Circulating oxidized LDL levels, current smoking and obesity in postmenopausal women. Atherosclerosis.

[132] Nunes J.M., Kruger A., Proal A., Kell D.B., Pretorius E. (2022). The Occurrence of Hyperactivated Platelets and Fibrinaloid Microclots in Myalgic Encephalomyelitis/Chronic Fatigue Syndrome (ME/CFS). Pharmaceuticals.

[133] Xiang M., Wu X., Jing H., Novakovic V.A., Shi J. (2023). The intersection of obesity and (long) COVID-19: Hypoxia, thrombotic inflammation, and vascular endothelial injury. Front. Cardiovasc. Med..

[134] Kell D.B., Laubscher G.J., Pretorius E. (2022). A central role for amyloid fibrin microclots in long COVID/PASC: Origins and therapeutic implications. Biochem. J..

[135] Bansal R., Gubbi S., Koch C.A. (2022). COVID-19 and chronic fatigue syndrome: An endocrine perspective. J. Clin. Transl. Endocrinol..

[136] Wheatland R. (2004). Molecular mimicry of ACTH in SARS—Implications for corticosteroid treatment and prophylaxis. Med. Hypotheses.

[137] Al-Hakeim H.K., Al-Rubaye H.T., Jubran A.S., Almulla A.F., Moustafa S.R., Maes M. (2023). Increased insulin resistance due to Long COVID is associated with depressive symptoms and partly predicted by the inflammatory response during acute infection. Braz. J. Psychiatry.

[138] Dodd S., Sominsky L., Siskind D., Bortolasci C.C., Carvalho A.F., Maes M., Walker A.J., Walder K., Yung A.R., Williams L.J. (2022). The role of metformin as a treatment for neuropsychiatric illness. Eur. Neuropsychopharmacol. J. Eur. Coll. Neuropsychopharmacol..

[139] Scherer P.E., Kirwan J.P., Rosen C.J. (2022). Post-acute sequelae of COVID-19: A metabolic perspective. eLife.

[140] Tan D.X., Reiter R.J. (2022). Mechanisms and clinical evidence to support melatonin’s use in severe COVID-19 patients to lower mortality. Life Sci..

[141] Cardinali D.P., Brown G.M., Pandi-Perumal S.R. (2022). Possible Application of Melatonin in Long COVID. Biomolecules.

[142] Etter M.M., Martins T.A., Kulsvehagen L., Pössnecker E., Duchemin W., Hogan S., Sanabria-Diaz G., Müller J., Chiappini A., Rychen J. (2022). Severe Neuro-COVID is associated with peripheral immune signatures, autoimmunity and neurodegeneration: A prospective cross-sectional study. Nat. Commun..

[143] Spudich S., Nath A. (2022). Nervous system consequences of COVID-19. Science.

[144] Leng A., Shah M., Ahmad S.A., Premraj L., Wildi K., Li Bassi G., Pardo C.A., Choi A., Cho S.M. (2023). Pathogenesis Underlying Neurological Manifestations of Long COVID Syndrome and Potential Therapeutics. Cells.

[145] Raj S.R., Bourne K.M., Stiles L.E., Miglis M.G., Cortez M.M., Miller A.J., Freeman R., Biaggioni I., Rowe P.C., Sheldon R.S. (2021). Postural orthostatic tachycardia syndrome (POTS): Priorities for POTS care and research from a 2019 National Institutes of Health Expert Consensus Meeting—Part 2. Auton. Neurosci. Basic Clin..

[146] Wallukat G., Hohberger B., Wenzel K., Fürst J., Schulze-Rothe S., Wallukat A., Hönicke A.S., Müller J. (2021). Functional autoantibodies against G-protein coupled receptors in patients with persistent Long-COVID-19 symptoms. J. Transl. Autoimmun..

[147] Sinaei R., Nejadbiglari H., Sinaei R., Zeinaly M., Pezeshki S., Jafari M. (2023). Finding positive SARS-CoV-2 RT-PCR in cerebrospinal fluid of two pediatric patients with severe COVID-19: A brief case report. BMC Pediatr..

[148] Jarius S., Pache F., Körtvelyessy P., Jelčić I., Stettner M., Franciotta D., Keller E., Neumann B., Ringelstein M., Senel M. (2022). Cerebrospinal fluid findings in COVID-19: A multicenter study of 150 lumbar punctures in 127 patients. J. Neuroinflamm..

[149] Díaz-Resendiz K.J.G., Benitez-Trinidad A.B., Covantes-Rosales C.E., Toledo-Ibarra G.A., Ortiz-Lazareno P.C., Girón-Pérez D.A., Bueno-Durán A.Y., Pérez-Díaz D.A., Barcelos-García R.G., Girón-Pérez M.I. (2022). Loss of mitochondrial membrane potential (ΔΨ(m)) in leucocytes as post-COVID-19 sequelae. J. Leukoc. Biol..

[150] Guntur V.P., Nemkov T., de Boer E., Mohning M.P., Baraghoshi D., Cendali F.I., San-Millán I., Petrache I., D’Alessandro A. (2022). Signatures of Mitochondrial Dysfunction and Impaired Fatty Acid Metabolism in Plasma of Patients with Post-Acute Sequelae of COVID-19 (PASC). Metabolites.

[151] Twomey R., DeMars J., Franklin K., Culos-Reed S.N., Weatherald J., Wrightson J.G. (2022). Chronic Fatigue and Postexertional Malaise in People Living with Long COVID: An Observational Study. Phys. Ther..

[152] Heerdt P.M., Shelley B., Singh I. (2022). Impaired systemic oxygen extraction long after mild COVID-19: Potential perioperative implications. Br. J. Anaesth..

[153] Paul B.D., Lemle M.D., Komaroff A.L., Snyder S.H. (2021). Redox imbalance links COVID-19 and myalgic encephalomyelitis/chronic fatigue syndrome. Proc. Natl. Acad. Sci. USA.

[154] de Boer E., Petrache I., Goldstein N.M., Olin J.T., Keith R.C., Modena B., Mohning M.P., Yunt Z.X., San-Millán I., Swigris J.J. (2022). Decreased Fatty Acid Oxidation and Altered Lactate Production during Exercise in Patients with Post-acute COVID-19 Syndrome. Am. J. Respir. Crit. Care Med..

[155] Terpos E., Ntanasis-Stathopoulos I., Elalamy I., Kastritis E., Sergentanis T.N., Politou M., Psaltopoulou T., Gerotziafas G., Dimopoulos M.A. (2020). Hematological findings and complications of COVID-19. Am. J. Hematol..

[156] Chen G., Wu D., Guo W., Cao Y., Huang D., Wang H., Wang T., Zhang X., Chen H., Yu H. (2020). Clinical and immunological features of severe and moderate coronavirus disease 2019. J. Clin. Investig..

[157] Kotecha T., Knight D.S., Razvi Y., Kumar K., Vimalesvaran K., Thornton G., Patel R., Chacko L., Brown J.T., Coyle C. (2021). Patterns of myocardial injury in recovered troponin-positive COVID-19 patients assessed by cardiovascular magnetic resonance. Eur. Heart J..

[158] Helms J., Tacquard C., Severac F., Leonard-Lorant I., Ohana M., Delabranche X., Merdji H., Clere-Jehl R., Schenck M., Fagot Gandet F. (2020). High risk of thrombosis in patients with severe SARS-CoV-2 infection: A multicenter prospective cohort study. Intensive Care Med..

[159] Karampela I., Christodoulatos G.S., Vallianou N., Tsilingiris D., Chrysanthopoulou E., Skyllas G., Antonakos G., Marinou I., Vogiatzakis E., Armaganidis A. (2022). Circulating Chemerin and Its Kinetics May Be a Useful Diagnostic and Prognostic Biomarker in Critically Ill Patients with Sepsis: A Prospective Study. Biomolecules.

[160] Karampela I., Christodoulatos G.S., Dalamaga M. (2019). The Role of Adipose Tissue and Adipokines in Sepsis: Inflammatory and Metabolic Considerations, and the Obesity Paradox. Curr. Obes. Rep..

[161] Dalamaga M., Karmaniolas K., Nikolaidou A., Papadavid E. (2008). Hypocalcemia, hypomagnesemia, and hypokalemia following hydrofluoric acid chemical injury. J. Burn Care Res. Off. Publ. Am. Burn Assoc..

[162] Karampela I., Kandri E., Antonakos G., Vogiatzakis E., Christodoulatos G.S., Nikolaidou A., Dimopoulos G., Armaganidis A., Dalamaga M. (2017). Kinetics of circulating fetuin-A may predict mortality independently from adiponectin, high molecular weight adiponectin and prognostic factors in critically ill patients with sepsis: A prospective study. J. Crit. Care.

[163] Shaw B.H., Stiles L.E., Bourne K., Green E.A., Shibao C.A., Okamoto L.E., Garland E.M., Gamboa A., Diedrich A., Raj V. (2019). The face of postural tachycardia syndrome—Insights from a large cross-sectional online community-based survey. J. Intern. Med..

[164] Houben-Wilke S., Goërtz Y.M., Delbressine J.M., Vaes A.W., Meys R., Machado F.V., van Herck M., Burtin C., Posthuma R., Franssen F.M. (2022). The Impact of Long COVID-19 on Mental Health: Observational 6-Month Follow-Up Study. JMIR Ment. Health.

[165] Naidu S.B., Shah A.J., Saigal A., Smith C., Brill S.E., Goldring J., Hurst J.R., Jarvis H., Lipman M., Mandal S. (2021). The high mental health burden of “Long COVID” and its association with on-going physical and respiratory symptoms in all adults discharged from hospital. Eur. Respir. J..

[166] Roca-Fernandez A., Wamil M., Telford A., Carapella V., Borlotti A., Monteiro D., Thomaides-Brears H., Kelly M., Dennis A., Banerjee R. (2023). Cardiac abnormalities in Long COVID 1-year post-SARS-CoV-2 infection. Open Heart.

[167] Hughes S.E., Haroon S., Subramanian A., McMullan C., Aiyegbusi O.L., Turner G.M., Jackson L., Davies E.H., Frost C., McNamara G. (2022). Development and validation of the symptom burden questionnaire for long COVID (SBQ-LC): Rasch analysis. BMJ.

[168] Jamal S.M., Landers D.B., Hollenberg S.M., Turi Z.G., Glotzer T.V., Tancredi J., Parrillo J.E. (2022). Prospective Evaluation of Autonomic Dysfunction in Post-Acute Sequela of COVID-19. J. Am. Coll. Cardiol..

[169] Stavileci B., Özdemir E., Özdemir B., Ereren E., Cengiz M. (2022). De-novo development of fragmented QRS during a six-month follow-up period in patients with COVID-19 disease and its cardiac effects. J. Electrocardiol..

[170] Grist J.T., Collier G.J., Walters H., Kim M., Chen M., Abu Eid G., Laws A., Matthews V., Jacob K., Cross S. (2022). Lung Abnormalities Detected with Hyperpolarized (129)Xe MRI in Patients with Long COVID. Radiology.

[171] Bateman L., Bested A.C., Bonilla H.F., Chheda B.V., Chu L., Curtin J.M., Dempsey T.T., Dimmock M.E., Dowell T.G., Felsenstein D. (2021). Myalgic Encephalomyelitis/Chronic Fatigue Syndrome: Essentials of Diagnosis and Management. Mayo Clin. Proc..

[172] Yong S.J., Halim A., Halim M., Liu S., Aljeldah M., Al Shammari B.R., Alwarthan S., Alhajri M., Alawfi A., Alshengeti A. (2023). Inflammatory and vascular biomarkers in post-COVID-19 syndrome: A systematic review and meta-analysis of over 20 biomarkers. Rev. Med. Virol..

[173] Yin J.X., Agbana Y.L., Sun Z.S., Fei S.W., Zhao H.Q., Zhou X.N., Chen J.H., Kassegne K. (2023). Increased interleukin-6 is associated with long COVID-19: A systematic review and meta-analysis. Infect. Dis. Poverty.

[174] Klein J., Wood J., Jaycox J., Lu P., Dhodapkar R.M., Gehlhausen J.R., Tabachnikova A., Tabacof L., Malik A.A., Kamath K. (2022). Distinguishing features of Long COVID identified through immune profiling. medRxiv.

[175] Haunhorst S., Bloch W., Javelle F., Krüger K., Baumgart S., Drube S., Lemhöfer C., Reuken P., Stallmach A., Müller M. (2022). A scoping review of regulatory T cell dynamics in convalescent COVID-19 patients—Indications for their potential involvement in the development of Long COVID?. Front. Immunol..

[176] Galán M., Vigón L., Fuertes D., Murciano-Antón M.A., Casado-Fernández G., Domínguez-Mateos S., Mateos E., Ramos-Martín F., Planelles V., Torres M. (2022). Persistent Overactive Cytotoxic Immune Response in a Spanish Cohort of Individuals with Long-COVID: Identification of Diagnostic Biomarkers. Front. Immunol..

[177] Utrero-Rico A., Ruiz-Ruigómez M., Laguna-Goya R., Arrieta-Ortubay E., Chivite-Lacaba M., González-Cuadrado C., Lalueza A., Almendro-Vazquez P., Serrano A., Aguado J.M. (2021). A Short Corticosteroid Course Reduces Symptoms and Immunological Alterations Underlying Long-COVID. Biomedicines.

[178] Patterson B.K., Guevara-Coto J., Yogendra R., Francisco E.B., Long E., Pise A., Rodrigues H., Parikh P., Mora J., Mora-Rodríguez R.A. (2021). Immune-Based Prediction of COVID-19 Severity and Chronicity Decoded Using Machine Learning. Front. Immunol..

[179] Swank Z., Senussi Y., Manickas-Hill Z., Yu X.G., Li J.Z., Alter G., Walt D.R. (2023). Persistent Circulating Severe Acute Respiratory Syndrome Coronavirus 2 Spike Is Associated with Post-acute Coronavirus Disease 2019 Sequelae. Clin. Infect. Dis..

[180] Natarajan A., Zlitni S., Brooks E.F., Vance S.E., Dahlen A., Hedlin H., Park R.M., Han A., Schmidtke D.T., Verma R. (2022). Gastrointestinal symptoms and fecal shedding of SARS-CoV-2 RNA suggest prolonged gastrointestinal infection. Med.

[181] Tejerina F., Catalan P., Rodriguez-Grande C., Adan J., Rodriguez-Gonzalez C., Muñoz P., Aldamiz T., Diez C., Perez L., Fanciulli C. (2022). Post-COVID-19 syndrome. SARS-CoV-2 RNA detection in plasma, stool, and urine in patients with persistent symptoms after COVID-19. BMC Infect. Dis..

[182] Zollner A., Koch R., Jukic A., Pfister A., Meyer M., Rössler A., Kimpel J., Adolph T.E., Tilg H. (2022). Postacute COVID-19 is Characterized by Gut Viral Antigen Persistence in Inflammatory Bowel Diseases. Gastroenterology.

[183] Yonker L.M., Gilboa T., Ogata A.F., Senussi Y., Lazarovits R., Boribong B.P., Bartsch Y.C., Loiselle M., Rivas M.N., Porritt R.A. (2021). Multisystem inflammatory syndrome in children is driven by zonulin-dependent loss of gut mucosal barrier. J. Clin. Investig..

[184] Files J.K., Sarkar S., Fram T.R., Boppana S., Sterrett S., Qin K., Bansal A., Long D.M., Sabbaj S., Kobie J.J. (2021). Duration of post-COVID-19 symptoms is associated with sustained SARS-CoV-2-specific immune responses. JCI Insight.

[185] Augustin M., Schommers P., Stecher M., Dewald F., Gieselmann L., Gruell H., Horn C., Vanshylla K., Cristanziano V.D., Osebold L. (2021). Post-COVID syndrome in non-hospitalised patients with COVID-19: A longitudinal prospective cohort study. Lancet Reg. Health. Eur..

[186] Blomberg B., Mohn K.G., Brokstad K.A., Zhou F., Linchausen D.W., Hansen B.A., Lartey S., Onyango T.B., Kuwelker K., Sævik M. (2021). Long COVID in a prospective cohort of home-isolated patients. Nat. Med..

[187] Peluso M.J., Deitchman A.N., Torres L., Iyer N.S., Munter S.E., Nixon C.C., Donatelli J., Thanh C., Takahashi S., Hakim J. (2021). Long-term SARS-CoV-2-specific immune and inflammatory responses in individuals recovering from COVID-19 with and without post-acute symptoms. Cell Rep..

[188] Peluso M.J., Deveau T.M., Munter S.E., Ryder D., Buck A., Beck-Engeser G., Chan F., Lu S., Goldberg S.A., Hoh R. (2023). Chronic viral coinfections differentially affect the likelihood of developing long COVID. J. Clin. Investig..

[189] Zubchenko S., Kril I., Nadizhko O., Matsyura O., Chopyak V. (2022). Herpesvirus infections and post-COVID-19 manifestations: A pilot observational study. Rheumatol. Int..

[190] Su Y., Yuan D., Chen D.G., Ng R.H., Wang K., Choi J., Li S., Hong S., Zhang R., Xie J. (2022). Multiple early factors anticipate post-acute COVID-19 sequelae. Cell.

[191] Gold J.E., Okyay R.A., Licht W.E., Hurley D.J. (2021). Investigation of Long COVID Prevalence and Its Relationship to Epstein-Barr Virus Reactivation. Pathogens.

[192] Muri J., Cecchinato V., Cavalli A., Shanbhag A.A., Matkovic M., Biggiogero M., Maida P.A., Moritz J., Toscano C., Ghovehoud E. (2023). Autoantibodies against chemokines post-SARS-CoV-2 infection correlate with disease course. Nat. Immunol..

[193] Bodansky A., Wang C.Y., Saxena A., Mitchell A., Takahashi S., Anglin K., Huang B., Hoh R., Lu S., Goldberg S.A. (2023). Autoantigen profiling reveals a shared post-COVID signature in fully recovered and Long COVID patients. medRxiv.

[194] Son K., Jamil R., Chowdhury A., Mukherjee M., Venegas C., Miyasaki K., Zhang K., Patel Z., Salter B., Yuen A.C.Y. (2023). Circulating anti-nuclear autoantibodies in COVID-19 survivors predict long COVID symptoms. Eur. Respir. J..

[195] Franke C., Boesl F., Goereci Y., Gerhard A., Schweitzer F., Schroeder M., Foverskov-Rasmussen H., Heine J., Quitschau A., Kandil F.I. (2023). Association of cerebrospinal fluid brain-binding autoantibodies with cognitive impairment in post-COVID-19 syndrome. Brain Behav. Immun..

[196] Peluso M.J., Mitchell A., Wang C.Y., Takahashi S., Hoh R., Tai V., Durstenfeld M.S., Hsue P.Y., Kelly J.D., Martin J.N. (2023). Low Prevalence of Interferon α Autoantibodies in People Experiencing Symptoms of Post-Coronavirus Disease 2019 (COVID-19) Conditions, or Long COVID. J. Infect. Dis..

[197] Rojas M., Rodríguez Y., Acosta-Ampudia Y., Monsalve D.M., Zhu C., Li Q.Z., Ramírez-Santana C., Anaya J.M. (2022). Autoimmunity is a hallmark of post-COVID syndrome. J. Transl. Med..

[198] Patel M.A., Knauer M.J., Nicholson M., Daley M., Van Nynatten L.R., Martin C., Patterson E.K., Cepinskas G., Seney S.L., Dobretzberger V. (2022). Elevated vascular transformation blood biomarkers in Long-COVID indicate angiogenesis as a key pathophysiological mechanism. Mol. Med..

[199] Haffke M., Freitag H., Rudolf G., Seifert M., Doehner W., Scherbakov N., Hanitsch L., Wittke K., Bauer S., Konietschke F. (2022). Endothelial dysfunction and altered endothelial biomarkers in patients with post-COVID-19 syndrome and chronic fatigue syndrome (ME/CFS). J. Transl. Med..

[200] Tong M., Yan X., Jiang Y., Jin Z., Zhu S., Zou L., Liu Y., Zheng Q., Chen G., Gu R. (2022). Endothelial Biomarkers in Patients Recovered from COVID-19 One Year after Hospital Discharge: A Cross-Sectional Study. Mediterr. J. Hematol. Infect. Dis..

[201] Constantinescu-Bercu A., Kessler A., de Groot R., Dragunaite B., Heightman M., Hillman T., Price L.C., Brennan E., Sivera R., Vanhoorelbeke K. (2023). Analysis of thrombogenicity under flow reveals new insights into the prothrombotic state of patients with post-COVID syndrome. J. Thromb. Haemost. JTH.

[202] Di Gennaro L., Valentini P., Sorrentino S., Ferretti M.A., De Candia E., Basso M., Lancellotti S., De Cristofaro R., De Rose C., Mariani F. (2022). Extended coagulation profile of children with Long COVID: A prospective study. Sci. Rep..

[203] Kruger A., Vlok M., Turner S., Venter C., Laubscher G.J., Kell D.B., Pretorius E. (2022). Proteomics of fibrin amyloid microclots in long COVID/post-acute sequelae of COVID-19 (PASC) shows many entrapped pro-inflammatory molecules that may also contribute to a failed fibrinolytic system. Cardiovasc. Diabetol..

[204] Pretorius E., Vlok M., Venter C., Bezuidenhout J.A., Laubscher G.J., Steenkamp J., Kell D.B. (2021). Persistent clotting protein pathology in Long COVID/Post-Acute Sequelae of COVID-19 (PASC) is accompanied by increased levels of antiplasmin. Cardiovasc. Diabetol..

[205] di Filippo L., Frara S., Nannipieri F., Cotellessa A., Locatelli M., Rovere Querini P., Giustina A. (2023). Low vitamin D levels are associated with Long COVID syndrome in COVID-19 survivors. J. Clin. Endocrinol. Metab..

[206] Mohamed Hussein A.A.R., Galal I., Amin M.T., Moshnib A.A., Makhlouf N.A., Makhlouf H.A., Abd-Elaal H.K., Kholief K.M.S., Abdel Tawab D.A., Kamal Eldin K.A. (2022). Prevalence of vitamin D deficiency among patients attending Post COVID-19 follow-up clinic: A cross-sectional study. Eur. Rev. Med. Pharmacol. Sci..

[207] Sunada N., Honda H., Nakano Y., Yamamoto K., Tokumasu K., Sakurada Y., Matsuda Y., Hasegawa T., Otsuka Y., Obika M. (2022). Hormonal trends in patients suffering from long COVID symptoms. Endocr. J..

[208] Townsend L., Dyer A.H., McCluskey P., O’Brien K., Dowds J., Laird E., Bannan C., Bourke N.M., Cheallaigh C.N., Byrne D.G. (2021). Investigating the Relationship between Vitamin D and Persistent Symptoms Following SARS-CoV-2 Infection. Nutrients.

[209] Captur G., Moon J.C., Topriceanu C.C., Joy G., Swadling L., Hallqvist J., Doykov I., Patel N., Spiewak J., Baldwin T. (2022). Plasma proteomic signature predicts who will get persistent symptoms following SARS-CoV-2 infection. EBioMedicine.

[210] López-Hernández Y., Oropeza-Valdez J.J., García Lopez D.A., Borrego J.C., Murgu M., Valdez J., López J.A., Monárrez-Espino J. (2023). Untargeted analysis in post-COVID-19 patients reveals dysregulated lipid pathways two years after recovery. Front. Mol. Biosci..

[211] López-Hernández Y., Aquino J.M., López D.A.G., Zheng J., Borrego J.C., Torres-Calzada C., Elizalde-Díaz J.P., Mandal R., Berjanskii M., Martínez-Martínez E. (2023). The plasma metabolome of long COVID-19 patients two years after infection. medRxiv.

[212] Cysique L.A., Jakabek D., Bracken S.G., Allen-Davidian Y., Heng B., Chow S., Dehhaghi M., Pires A.S., Darley D.R., Byrne A. (2022). Post-acute COVID-19 cognitive impairment and decline uniquely associate with kynurenine pathway activation: A longitudinal observational study. medRxiv.

[213] Zhang D., Zhou Y., Ma Y., Chen P., Tang J., Yang B., Li H., Liang M., Xue Y., Liu Y. (2023). Gut Microbiota Dysbiosis Correlates with Long COVID-19 at One-Year After Discharge. J. Korean Med. Sci..

[214] Liu Q., Mak J.W.Y., Su Q., Yeoh Y.K., Lui G.C., Ng S.S.S., Zhang F., Li A.Y.L., Lu W., Hui D.S. (2022). Gut microbiota dynamics in a prospective cohort of patients with post-acute COVID-19 syndrome. Gut.

[215] Apple A.C., Oddi A., Peluso M.J., Asken B.M., Henrich T.J., Kelly J.D., Pleasure S.J., Deeks S.G., Allen I.E., Martin J.N. (2022). Risk factors and abnormal cerebrospinal fluid associate with cognitive symptoms after mild COVID-19. Ann. Clin. Transl. Neurol..

[216] Guasp M., Muñoz-Sánchez G., Martínez-Hernández E., Santana D., Carbayo Á., Naranjo L., Bolós U., Framil M., Saiz A., Balasa M. (2022). CSF Biomarkers in COVID-19 Associated Encephalopathy and Encephalitis Predict Long-Term Outcome. Front. Immunol..

[217] Bonnet B., Cosme J., Dupuis C., Coupez E., Adda M., Calvet L., Fabre L., Saint-Sardos P., Bereiziat M., Vidal M. (2021). Severe COVID-19 is characterized by the co-occurrence of moderate cytokine inflammation and severe monocyte dysregulation. EBioMedicine.

[218] Montazersaheb S., Hosseiniyan Khatibi S.M., Hejazi M.S., Tarhriz V., Farjami A., Ghasemian Sorbeni F., Farahzadi R., Ghasemnejad T. (2022). COVID-19 infection: An overview on cytokine storm and related interventions. Virol. J..

[219] Low R.N., Low R.J., Akrami A. (2023). A review of cytokine-based pathophysiology of Long COVID symptoms. Front. Med..

[220] Peluso M.J., Lu S., Tang A.F., Durstenfeld M.S., Ho H.E., Goldberg S.A., Forman C.A., Munter S.E., Hoh R., Tai V. (2021). Markers of Immune Activation and Inflammation in Individuals with Postacute Sequelae of Severe Acute Respiratory Syndrome Coronavirus 2 Infection. J. Infect. Dis..

[221] Littlefield K.M., Watson R.O., Schneider J.M., Neff C.P., Yamada E., Zhang M., Campbell T.B., Falta M.T., Jolley S.E., Fontenot A.P. (2022). SARS-CoV-2-specific T cells associate with inflammation and reduced lung function in pulmonary post-acute sequalae of SARS-CoV-2. PLoS Pathog..

[222] Cortellini A., Gennari A., Pommeret F., Patel G., Newsom-Davis T., Bertuzzi A., Viladot M., Aguilar-Company J., Mirallas O., Felip E. (2022). COVID-19 Sequelae and the Host Proinflammatory Response: An Analysis from the OnCovid Registry. J. Natl. Cancer Inst..

[223] Hu S.J., Weng Z.C. (1988). [Influence of stimulation of the skin receptive field on evoked discharges of the polymodal nociceptors in rats]. Sheng Li Xue Bao.

[224] Queiroz M.A.F., Neves P., Lima S.S., Lopes J.D.C., Torres M., Vallinoto I., Bichara C.D.A., Dos Santos E.F., de Brito M., da Silva A.L.S. (2022). Cytokine Profiles Associated with Acute COVID-19 and Long COVID-19 Syndrome. Front. Cell. Infect. Microbiol..

[225] Fernández-Castañeda A., Lu P., Geraghty A.C., Song E., Lee M.H., Wood J., Yalçın B., Taylor K.R., Dutton S., Acosta-Alvarez L. (2022). Mild respiratory SARS-CoV-2 infection can cause multi-lineage cellular dysregulation and myelin loss in the brain. bioRxiv.

[226] Venkataramani V., Winkler F. (2022). Cognitive Deficits in Long COVID-19. New Engl. J. Med..

[227] da Silva R., de Sarges K.M.L., Cantanhede M.H.D., da Costa F.P., Dos Santos E.F., Rodrigues F.B.B., de Nazaré do Socorro de Almeida Viana M., de Meira Leite M., da Silva A.L.S., de Brito M.T.M. (2023). Thrombophilia and Immune-Related Genetic Markers in Long COVID. Viruses.

[228] Hornig M., Montoya J.G., Klimas N.G., Levine S., Felsenstein D., Bateman L., Peterson D.L., Gottschalk C.G., Schultz A.F., Che X. (2015). Distinct plasma immune signatures in ME/CFS are present early in the course of illness. Sci. Adv..

[229] Glynne P., Tahmasebi N., Gant V., Gupta R. (2022). Long COVID following mild SARS-CoV-2 infection: Characteristic T cell alterations and response to antihistamines. J. Investig. Med..

[230] Cheung C.C.L., Goh D., Lim X., Tien T.Z., Lim J.C.T., Lee J.N., Tan B., Tay Z.E.A., Wan W.Y., Chen E.X. (2022). Residual SARS-CoV-2 viral antigens detected in GI and hepatic tissues from five recovered patients with COVID-19. Gut.

[231] Gaebler C., Wang Z., Lorenzi J.C.C., Muecksch F., Finkin S., Tokuyama M., Cho A., Jankovic M., Schaefer-Babajew D., Oliveira T.Y. (2021). Evolution of antibody immunity to SARS-CoV-2. Nature.

[232] Sigal G.B., Novak T., Mathew A., Chou J., Zhang Y., Manjula N., Bathala P., Joe J., Padmanabhan N., Romero D. (2022). Measurement of Severe Acute Respiratory Syndrome Coronavirus 2 Antigens in Plasma of Pediatric Patients with Acute Coronavirus Disease 2019 or Multisystem Inflammatory Syndrome in Children Using an Ultrasensitive and Quantitative Immunoassay. Clin. Infect. Dis..

[233] Yang C., Zhao H., Espín E., Tebbutt S.J. (2023). Association of SARS-CoV-2 infection and persistence with long COVID. Lancet. Respir. Med..

[234] Barouch D.H. (2022). COVID-19 Vaccines—Immunity, Variants, Boosters. New Engl. J. Med..

[235] Banko A., Miljanovic D., Cirkovic A. (2023). Systematic review with meta-analysis of active herpesvirus infections in patients with COVID-19: Old players on the new field. Int. J. Infect. Dis..

[236] Manoharan S., Ying L.Y. (2023). Epstein Barr Virus Reactivation during COVID-19 Hospitalization Significantly Increased Mortality/Death in SARS-CoV-2(+)/EBV(+) than SARS-CoV-2(+)/EBV(−) Patients: A Comparative Meta-Analysis. Int. J. Clin. Pract..

[237] Ruiz-Pablos M., Paiva B., Montero-Mateo R., Garcia N., Zabaleta A. (2021). Epstein-Barr Virus and the Origin of Myalgic Encephalomyelitis or Chronic Fatigue Syndrome. Front. Immunol..

[238] Apostolou E., Rizwan M., Moustardas P., Sjögren P., Bertilson B.C., Bragée B., Polo O., Rosén A. (2022). Saliva antibody-fingerprint of reactivated latent viruses after mild/asymptomatic COVID-19 is unique in patients with myalgic-encephalomyelitis/chronic fatigue syndrome. Front. Immunol..

[239] Suurmond J., Diamond B. (2015). Autoantibodies in systemic autoimmune diseases: Specificity and pathogenicity. J. Clin. Investig..

[240] Knight J.S., Caricchio R., Casanova J.L., Combes A.J., Diamond B., Fox S.E., Hanauer D.A., James J.A., Kanthi Y., Ladd V. (2021). The intersection of COVID-19 and autoimmunity. J. Clin. Investig..

[241] Wang E.Y., Mao T., Klein J., Dai Y., Huck J.D., Jaycox J.R., Liu F., Zhou T., Israelow B., Wong P. (2021). Diverse functional autoantibodies in patients with COVID-19. Nature.

[242] Bastard P., Gervais A., Le Voyer T., Rosain J., Philippot Q., Manry J., Michailidis E., Hoffmann H.H., Eto S., Garcia-Prat M. (2021). Autoantibodies neutralizing type I IFNs are present in ~4% of uninfected individuals over 70 years old and account for ~20% of COVID-19 deaths. Sci. Immunol..

[243] Manry J., Bastard P., Gervais A., Le Voyer T., Rosain J., Philippot Q., Michailidis E., Hoffmann H.H., Eto S., Garcia-Prat M. (2022). The risk of COVID-19 death is much greater and age dependent with type I IFN autoantibodies. Proc. Natl. Acad. Sci. USA.

[244] Eto S., Nukui Y., Tsumura M., Nakagama Y., Kashimada K., Mizoguchi Y., Utsumi T., Taniguchi M., Sakura F., Noma K. (2022). Neutralizing Type I Interferon Autoantibodies in Japanese Patients with Severe COVID-19. Res. Sq..

[245] Zhang Q., Bastard P., Cobat A., Casanova J.L. (2022). Human genetic and immunological determinants of critical COVID-19 pneumonia. Nature.

[246] Peluso M.J., Thomas I.J., Munter S.E., Deeks S.G., Henrich T.J. (2022). Lack of Antinuclear Antibodies in Convalescent Coronavirus Disease 2019 Patients with Persistent Symptoms. Clin. Infect. Dis. Off. Publ. Infect. Dis. Soc. Am..

[247] Arthur J.M., Forrest J.C., Boehme K.W., Kennedy J.L., Owens S., Herzog C., Liu J., Harville T.O. (2021). Development of ACE2 autoantibodies after SARS-CoV-2 infection. PLoS ONE.

[248] Fogarty H., Townsend L., Morrin H., Ahmad A., Comerford C., Karampini E., Englert H., Byrne M., Bergin C., O’Sullivan J.M. (2021). Persistent endotheliopathy in the pathogenesis of long COVID syndrome. J. Thromb. Haemost. JTH.

[249] Jarrott B., Head R., Pringle K.G., Lumbers E.R., Martin J.H. (2022). “LONG COVID”-A hypothesis for understanding the biological basis and pharmacological treatment strategy. Pharmacol. Res. Perspect..

[250] Fan B.E., Wong S.W., Sum C.L.L., Lim G.H., Leung B.P., Tan C.W., Ramanathan K., Dalan R., Cheung C., Lim X.R. (2022). Hypercoagulability, endotheliopathy, and inflammation approximating 1 year after recovery: Assessing the long-term outcomes in COVID-19 patients. Am. J. Hematol..

[251] Kell D.B., Pretorius E. (2022). The potential role of ischaemia-reperfusion injury in chronic, relapsing diseases such as rheumatoid arthritis, Long COVID, and ME/CFS: Evidence, mechanisms, and therapeutic implications. Biochem. J..

[252] Pretorius E., Venter C., Laubscher G.J., Kotze M.J., Oladejo S.O., Watson L.R., Rajaratnam K., Watson B.W., Kell D.B. (2022). Prevalence of symptoms, comorbidities, fibrin amyloid microclots and platelet pathology in individuals with Long COVID/Post-Acute Sequelae of COVID-19 (PASC). Cardiovasc. Diabetol..

[253] Du W.N., Zhang Y., Yu Y., Zhang R.M. (2021). D-dimer levels is associated with severe COVID-19 infections: A meta-analysis. Int. J. Clin. Pract..

[254] Rushworth R.L., Torpy D.J., Falhammar H. (2019). Adrenal Crisis. New Engl. J. Med..

[255] Demitrack M.A., Dale J.K., Straus S.E., Laue L., Listwak S.J., Kruesi M.J., Chrousos G.P., Gold P.W. (1991). Evidence for impaired activation of the hypothalamic-pituitary-adrenal axis in patients with chronic fatigue syndrome. J. Clin. Endocrinol. Metab..

[256] Lin Y.J., Ko Y.C., Chow L.H., Hsiao F.J., Liu H.Y., Wang P.N., Chen W.T. (2021). Salivary cortisol is associated with cognitive changes in patients with fibromyalgia. Sci. Rep..

[257] Leow M.K., Kwek D.S., Ng A.W., Ong K.C., Kaw G.J., Lee L.S. (2005). Hypocortisolism in survivors of severe acute respiratory syndrome (SARS). Clin. Endocrinol..

[258] Choy K.W. (2020). Cortisol concentrations and mortality from COVID-19. Lancet. Diabetes Endocrinol..

[259] Kedor C., Freitag H., Meyer-Arndt L., Wittke K., Hanitsch L.G., Zoller T., Steinbeis F., Haffke M., Rudolf G., Heidecker B. (2022). A prospective observational study of post-COVID-19 chronic fatigue syndrome following the first pandemic wave in Germany and biomarkers associated with symptom severity. Nat. Commun..

[260] Bonilla H., Quach T.C., Tiwari A., Bonilla A.E., Miglis M., Yang P.C., Eggert L.E., Sharifi H., Horomanski A., Subramanian A. (2023). Myalgic Encephalomyelitis/Chronic Fatigue Syndrome is common in post-acute sequelae of SARS-CoV-2 infection (PASC): Results from a post-COVID-19 multidisciplinary clinic. Front. Neurol..

[261] Dalamaga M., Christodoulatos G.S. (2015). Adiponectin as a biomarker linking obesity and adiposopathy to hematologic malignancies. Horm. Mol. Biol. Clin. Investig..

[262] Dalamaga M., Karmaniolas K., Chamberland J., Nikolaidou A., Lekka A., Dionyssiou-Asteriou A., Mantzoros C.S. (2013). Higher fetuin-A, lower adiponectin and free leptin levels mediate effects of excess body weight on insulin resistance and risk for myelodysplastic syndrome. Metabolism.

[263] Dalamaga M., Crotty B.H., Fargnoli J., Papadavid E., Lekka A., Triantafilli M., Karmaniolas K., Migdalis I., Dionyssiou-Asteriou A., Mantzoros C.S. (2010). B-cell chronic lymphocytic leukemia risk in association with serum leptin and adiponectin: A case-control study in Greece. Cancer Causes Control. CCC.

[264] Dalamaga M., Karmaniolas K., Nikolaidou A., Chamberland J., Hsi A., Dionyssiou-Asteriou A., Mantzoros C.S. (2008). Adiponectin and resistin are associated with risk for myelodysplastic syndrome, independently from the insulin-like growth factor-I (IGF-I) system. Eur. J. Cancer.

[265] Dalamaga M., Nikolaidou A., Karmaniolas K., Hsi A., Chamberland J., Dionyssiou-Asteriou A., Mantzoros C.S. (2007). Circulating adiponectin and leptin in relation to myelodysplastic syndrome: A case-control study. Oncology.

[266] Marouga A., Dalamaga M., Kastania A.N., Antonakos G., Thrasyvoulides A., Kontelia G., Dimas C., Vlahakos D.V. (2013). Correlates of serum resistin in elderly, non-diabetic patients with chronic kidney disease. Clin. Lab..

[267] Papadavid E., Gazi S., Dalamaga M., Stavrianeas N., Ntelis V. (2008). Palmoplantar and scalp psoriasis occurring during anti-tumour necrosis factor-alpha therapy: A case series of four patients and guidelines for management. J. Eur. Acad. Dermatol. Venereol..

[268] Papadavid E., Vlami K., Dalamaga M., Giatrakou S., Theodoropoulos K., Gyftopoulos S., Stavrianeas N., Papiris S., Rigopoulos D. (2013). Sleep apnea as a comorbidity in obese psoriasis patients: A cross-sectional study. Do psoriasis characteristics and metabolic parameters play a role?. J. Eur. Acad. Dermatol. Venereol..

[269] Papadavid E., Dalamaga M., Vlami K., Koumaki D., Gyftopoulos S., Christodoulatos G.S., Papiris S., Rigopoulos D. (2017). Psoriasis is associated with risk of obstructive sleep apnea independently from metabolic parameters and other comorbidities: A large hospital-based case-control study. Sleep Breath. Schlaf Atm..

[270] Pavlidou A., Dalamaga M., Kroupis C., Konstantoudakis G., Belimezi M., Athanasas G., Dimas K. (2011). Survivin isoforms and clinicopathological characteristics in colorectal adenocarcinomas using real-time qPCR. World J. Gastroenterol..

[271] Dalamaga M., Karmaniolas K., Lekka A., Antonakos G., Thrasyvoulides A., Papadavid E., Spanos N., Dionyssiou-Asteriou A. (2010). Platelet markers correlate with glycemic indices in diabetic, but not diabetic-myelodysplastic patients with normal platelet count. Dis. Mrk..

[272] Bramante C.T., Buse J.B., Liebovitz D., Nicklas J., Puskarich M.A., Cohen K., Belani H., Anderson B., Huling J.D., Tignanelli C. (2022). Outpatient treatment of COVID-19 with metformin, ivermectin, and fluvoxamine and the development of Long COVID over 10-month follow-up. medRxiv.

[273] Dissanayake H.A., de Silva N.L., Sumanatilleke M., de Silva S.D.N., Gamage K.K.K., Dematapitiya C., Kuruppu D.C., Ranasinghe P., Pathmanathan S., Katulanda P. (2022). Prognostic and Therapeutic Role of Vitamin D in COVID-19: Systematic Review and Meta-analysis. J. Clin. Endocrinol. Metab..

[274] Akbar M.R., Wibowo A., Pranata R., Setiabudiawan B. (2021). Corrigendum: Low Serum 25-hydroxyvitamin D (Vitamin D) Level Is Associated with Susceptibility to COVID-19, Severity, and Mortality: A Systematic Review and Meta-Analysis. Front. Nutr..

[275] Bilezikian J.P., Bikle D., Hewison M., Lazaretti-Castro M., Formenti A.M., Gupta A., Madhavan M.V., Nair N., Babalyan V., Hutchings N. (2020). MECHANISMS IN ENDOCRINOLOGY: Vitamin D and COVID-19. Eur. J. Endocrinol..

[276] Moukayed M. (2023). A Narrative Review on the Potential Role of Vitamin D(3) in the Prevention, Protection, and Disease Mitigation of Acute and Long COVID-19. Curr. Nutr. Rep..

[277] Bader-Larsen K.S., Larson E.A., Dalamaga M., Magkos F. (2021). A Narrative Review of the Safety of Anti-COVID-19 Nutraceuticals for Patients with Cancer. Cancers.

[278] Garcia M., Seelaender M., Sotiropoulos A., Coletti D., Lancha A.H. (2019). Vitamin D, muscle recovery, sarcopenia, cachexia, and muscle atrophy. Nutrition.

[279] Gáll Z., Székely O. (2021). Role of Vitamin D in Cognitive Dysfunction: New Molecular Concepts and Discrepancies between Animal and Human Findings. Nutrients.

[280] Dalamaga M., Muscogiuri G., Paganitsa G., Parvouleskou G., Syriou V., Karagkoynis P., Stratigou T., Vallianou N., Christodoulatos G.S., Karampela I. (2021). Adherence to the Mediterranean diet is an independent predictor of circulating vitamin D levels in normal weight and non-smoker adults: An observational cross-sectional study. Int. J. Food Sci. Nutr..

[281] Jolliffe D.A., Camargo C.A., Sluyter J.D., Aglipay M., Aloia J.F., Ganmaa D., Bergman P., Bischoff-Ferrari H.A., Borzutzky A., Damsgaard C.T. (2021). Vitamin D supplementation to prevent acute respiratory infections: A systematic review and meta-analysis of aggregate data from randomised controlled trials. Lancet. Diabetes Endocrinol..

[282] Mirhosseini N., Rainsbury J., Kimball S.M. (2018). Vitamin D Supplementation, Serum 25(OH)D Concentrations and Cardiovascular Disease Risk Factors: A Systematic Review and Meta-Analysis. Front. Cardiovasc. Med..

[283] Karampela I., Sakelliou A., Vallianou N., Christodoulatos G.S., Magkos F., Dalamaga M. (2021). Vitamin D and Obesity: Current Evidence and Controversies. Curr. Obes. Rep..

[284] Fernandes A.L., Sales L.P., Santos M.D., Caparbo V.F., Murai I.H., Pereira R.M.R. (2022). Persistent or new symptoms 1 year after a single high dose of vitamin D(3) in patients with moderate to severe COVID-19. Front. Nutr..

[285] Galluzzo V., Ciciarello F., Tosato M., Zazzara M.B., Pais C., Savera G., Calvani R., Picca A., Marzetti E., Landi F. (2022). Association between vitamin D status and physical performance in COVID-19 survivors: Results from the Gemelli against COVID-19 post-acute care project. Mech. Ageing Dev..

[286] Bruzzone C., Conde R., Embade N., Mato J.M., Millet O. (2023). Metabolomics as a powerful tool for diagnostic, pronostic and drug intervention analysis in COVID-19. Front. Mol. Biosci..

[287] König R.S., Albrich W.C., Kahlert C.R., Bahr L.S., Löber U., Vernazza P., Scheibenbogen C., Forslund S.K. (2021). The Gut Microbiome in Myalgic Encephalomyelitis (ME)/Chronic Fatigue Syndrome (CFS). Front. Immunol..

[288] Vallianou N.G., Kounatidis D., Tsilingiris D., Panagopoulos F., Christodoulatos G.S., Evangelopoulos A., Karampela I., Dalamaga M. (2023). The Role of Next-Generation Probiotics in Obesity and Obesity-Associated Disorders: Current Knowledge and Future Perspectives. Int. J. Mol. Sci..

[289] Papavasileiou G., Tsilingiris D., Spyrou N., Vallianou N.G., Karampela I., Magkos F., Dalamaga M. (2023). Obesity and main urologic cancers: Current systematic evidence, novel biological mechanisms, perspectives and challenges. Semin. Cancer Biol..

[290] Yeoh Y.K., Zuo T., Lui G.C., Zhang F., Liu Q., Li A.Y., Chung A.C., Cheung C.P., Tso E.Y., Fung K.S. (2021). Gut microbiota composition reflects disease severity and dysfunctional immune responses in patients with COVID-19. Gut.

[291] Zhang F., Lau R.I., Liu Q., Su Q., Chan F.K.L., Ng S.C. (2023). Gut microbiota in COVID-19: Key microbial changes, potential mechanisms and clinical applications. Nat. Rev. Gastroenterol. Hepatol..

[292] de Almeida V.M., Engel D., Ricci M.F., Cruz C.S., Lopes I.S., Alves D.A., Auriol M.D., Magalhães J., Zuccoli G.S., Smith B.J. (2022). Gut microbiota from patients with mild COVID-19 cause alterations in mice that resemble post-COVID syndrome. Res. Sq..

[293] Dalamaga M., Karmaniolas K., Arsenis G., Pantelaki M., Daskalopoulou K., Papadavid E., Migdalis I. (2008). Cedecea lapagei bacteremia following cement-related chemical burn injury. Burns.

[294] Papadavid E., Dalamaga M., Kapniari I., Pantelidaki E., Papageorgiou S., Pappa V., Tsirigotis P., Dervenoulas I., Stavrianeas N., Rigopoulos D. (2012). Lobomycosis: A case from Southeastern Europe and review of the literature. J. Dermatol. Case Rep..

[295] Giron L.B., Peluso M.J., Ding J., Kenny G., Zilberstein N.F., Koshy J., Hong K.Y., Rasmussen H., Miller G.E., Bishehsari F. (2022). Markers of fungal translocation are elevated during post-acute sequelae of SARS-CoV-2 and induce NF-κB signaling. JCI Insight.

[296] Domingues R.B., Leite F., Senne C. (2022). Cerebrospinal fluid analysis in patients with COVID-19-associated central nervous system manifestations: A systematic review. Arq. De Neuro-Psiquiatr..

[297] Perrin P., Collongues N., Baloglu S., Bedo D., Bassand X., Lavaux T., Gautier-Vargas G., Keller N., Kremer S., Fafi-Kremer S. (2021). Cytokine release syndrome-associated encephalopathy in patients with COVID-19. Eur. J. Neurol..

[298] Chaumont H., Kaczorowski F., San-Galli A., Michel P.P., Tressières B., Roze E., Quadrio I., Lannuzel A. (2023). Cerebrospinal fluid biomarkers in SARS-CoV-2 patients with acute neurological syndromes. Rev. Neurol..

[299] Guillot F., Garcia A., Salou M., Brouard S., Laplaud D.A., Nicot A.B. (2015). Transcript analysis of laser capture microdissected white matter astrocytes and higher phenol sulfotransferase 1A1 expression during autoimmune neuroinflammation. J. Neuroinflammation.

[300] Peluso M.J., Sans H.M., Forman C.A., Nylander A.N., Ho H.E., Lu S., Goldberg S.A., Hoh R., Tai V., Munter S.E. (2022). Plasma Markers of Neurologic Injury and Inflammation in People with Self-Reported Neurologic Postacute Sequelae of SARS-CoV-2 Infection. Neurol. (R) Neuroimmunol. Neuroinflamm..

[301] Magdy R., Eid R.A., Fathy W., Abdel-Aziz M.M., Ibrahim R.E., Yehia A., Sheemy M.S., Hussein M. (2022). Characteristics and Risk Factors of Persistent Neuropathic Pain in Recovered COVID-19 Patients. Pain Med..

[302] Peluso M.J., Deeks S.G., Mustapic M., Kapogiannis D., Henrich T.J., Lu S., Goldberg S.A., Hoh R., Chen J.Y., Martinez E.O. (2022). SARS-CoV-2 and Mitochondrial Proteins in Neural-Derived Exosomes of COVID-19. Ann. Neurol..

[303] Wu J., Tang L., Ma Y., Li Y., Zhang D., Li Q., Mei H., Hu Y. (2021). Immunological Profiling of COVID-19 Patients with Pulmonary Sequelae. mBio.

[304] Davis H.E., Assaf G.S., McCorkell L., Wei H., Low R.J., Re’em Y., Redfield S., Austin J.P., Akrami A. (2021). Characterizing long COVID in an international cohort: 7 months of symptoms and their impact. EClinicalMedicine.

[305] Apostolo D., D’Onghia D., Tonello S., Minisini R., Baricich A., Gramaglia C., Patrucco F., Zeppegno P., Acquaviva A., Balbo P.E. (2023). Decreased Gas6 and sAxl Plasma Levels Are Associated with Hair Loss in COVID-19 Survivors. Int. J. Mol. Sci..

[306] Haslam A., Olivier T., Prasad V. (2023). The definition of long COVID used in interventional studies. Eur. J. Clin. Investig..

[307] Fawzy N.A., Abou Shaar B., Taha R.M., Arabi T.Z., Sabbah B.N., Alkodaymi M.S., Omrani O.A., Makhzoum T., Almahfoudh N.E., Al-Hammad Q.A. (2023). A systematic review of trials currently investigating therapeutic modalities for post-acute COVID-19 syndrome and registered on WHO International Clinical Trials Platform. Clin. Microbiol. Infect..

[308] Greenhalgh T., Sivan M., Delaney B., Evans R., Milne R. (2022). Long COVID-an update for primary care. BMJ.

[309] Chadda K.R., Blakey E.E., Huang C.L., Jeevaratnam K. (2022). Long COVID-19 and Postural Orthostatic Tachycardia Syndrome—Is Dysautonomia to Be Blamed?. Front. Cardiovasc. Med..

[310] Geng L.N., Bonilla H.F., Shafer R.W., Miglis M.G., Yang P.C. (2023). The Use of Nirmatrelvir-ritonavir in a Case of Breakthrough Long COVID. Explor. Res. Hypothesis Med..

[311] Xie Y., Choi T., Al-Aly Z. (2023). Association of Treatment with Nirmatrelvir and the Risk of Post-COVID-19 Condition. JAMA Intern. Med..

[312] Bajema K.L., Berry K., Streja E., Rajeevan N., Li Y., Yan L., Cunningham F., Hynes D.M., Rowneki M., Bohnert A. (2022). Effectiveness of COVID-19 treatment with nirmatrelvir-ritonavir or molnupiravir among U.S. Veterans: Target trial emulation studies with one-month and six-month outcomes. medRxiv.

[313] Durstenfeld M.S., Peluso M.J., Lin F., Peyser N.D., Isasi C., Carton T.W., Henrich T.J., Deeks S.G., Olgin J.E., Pletcher M.J. (2023). Association of Nirmatrelvir/Ritonavir Treatment with Long COVID Symptoms in an Online Cohort of Non-Hospitalized Individuals Experiencing Breakthrough SARS-CoV-2 Infection in the Omicron Era. medRxiv.

[314] Parthasarathy H., Tandel D., Siddiqui A.H., Harshan K.H. (2022). Metformin suppresses SARS-CoV-2 in cell culture. Virus Res..

[315] Ventura-López C., Cervantes-Luevano K., Aguirre-Sánchez J.S., Flores-Caballero J.C., Alvarez-Delgado C., Bernaldez-Sarabia J., Sánchez-Campos N., Lugo-Sánchez L.A., Rodríguez-Vázquez I.C., Sander-Padilla J.G. (2022). Treatment with metformin glycinate reduces SARS-CoV-2 viral load: An in vitro model and randomized, double-blind, Phase IIb clinical trial. Biomed. Pharmacother. Biomed. Pharmacother..

[316] Daou N., Viader A., Cokol M., Nitzel A., Chakravarthy M.V., Afeyan R., Tramontin T., Marukian S., Hamill M.J. (2021). A novel, multitargeted endogenous metabolic modulator composition impacts metabolism, inflammation, and fibrosis in nonalcoholic steatohepatitis-relevant primary human cell models. Sci. Rep..

[317] Finnigan L.E.M., Cassar M.P., Koziel M.J., Pradines J., Lamlum H., Azer K., Kirby D., Montgomery H., Neubauer S., Valkovič L. (2023). Efficacy and tolerability of an endogenous metabolic modulator (AXA1125) in fatigue-predominant long COVID: A single-centre, double-blind, randomised controlled phase 2a pilot study. eClinicalMedicine.

[318] https://clinicaltrials.gov/.

[319] Kritis P., Karampela I., Kokoris S., Dalamaga M. (2020). The combination of bromelain and curcumin as an immune-boosting nutraceutical in the prevention of severe COVID-19. Metab. Open.

